# FGF21, a modulator of astrocyte reactivity, protects against ischemic brain injury through anti-inflammatory and neurotrophic pathways

**DOI:** 10.1038/s41401-024-01462-x

**Published:** 2025-02-28

**Authors:** Dong-xue Wang, Wen-ting Huang, Jun-feng Shi, Fei Liu, Wen-yi Jiang, Ke-yang Chen, Shu-yang Zhang, Xiao-kun Li, Li Lin

**Affiliations:** School of Pharmaceutical Sciences, Wenzhou Medical University, Wenzhou, Zhejiang 325035 China

**Keywords:** FGF21, transient middle cerebral artery occlusion (tMCAO), astrocyte reactivity, leukocyte infiltration, neuroinflammation

## Abstract

Ischemic stroke is a frequent cause of mortality and disability, and astrocyte reactivity is closely associated with injury outcomes. Fibroblast growth factor 21 (FGF21), an endogenous regulator, has been shown to perform pleiotropic functions in central nervous system (CNS) disorders. However, studies on neurological diseases have paid little attention to the effects and detailed mechanisms of FGF21 in astrocytes. Here, we found elevated serum levels of FGF21 in stroke patients and transient middle cerebral artery occlusion (tMCAO) mice. In the peri-infarct cortex, microglia and astrocytes serve as sources of FGF21 in addition to neurons. MRI and neurobehavioral assessments of wild-type (WT) and FGF21^−/−^ tMCAO model mice revealed a deteriorated consequence of the loss of FGF21, with exacerbated brain infarction and neurological deficits. Additionally, combined with the pharmacological treatment of WT mice with recombinant human FGF21 (rhFGF21) after tMCAO, FGF21 was identified to suppress astrocytic activation and astrocyte-mediated inflammatory responses after brain ischemia and participated in controlling the infiltration of peripheral inflammatory cells (including macrophages, neutrophils, monocytes, and T cells) by modulating chemokines expression (such as Ccl3, Cxcl1, and Cxcl2) in astrocytes. Furthermore, rhFGF21 was shown to boost the production of neurotrophic factors (BDNF and NGF) in astrocytes, and by which rescued neuronal survival and promoted synaptic protein expression (postsynaptic density protein-95 (PSD-95), synaptotagmin 1 (SYT1), and synaptophysin) in neurons after ischemic injury. Overall, our findings implicate that FGF21 acts as a suppressor of astrocyte activation, and exerts anti-inflammatory and neurotrophic effects after ischemic brain injury through its action on astrocytes, offering an alternative therapeutic target.

## Introduction

Ischemic stroke is a lethal condition caused by an arterial embolism that interrupts blood flow through the cerebral artery and is a frequent cause of mortality and disability [1]. Cessation of blood circulation results in a deficiency of oxygen and energy, and consequently leads to devastating damage to intricate neuronal circuits and the impairment of cognitive and sensorimotor function. Identifying the cellular and molecular mechanisms that underlie functional recovery may yield novel therapeutic approaches to promote neuronal repair, improve long-term outcomes, and ease the socioeconomic burden of stroke [2].

Astrocytes, the most abundant glial cells in the central nervous system (CNS), play a pivotal role in maintaining the essential functions of brain development [3]. They provide trophic support for neurons and structural support for the blood-brain barrier (BBB); guide the formation, maturation, and elimination of synapses; and intervene the uptake and recycling of neurotransmitters [4]. In response to pathogenic stimuli, ranging from injury, infection, to neurodegenerative disease, astrocytes enter a reactive state (reactive astrocytes) with a high level of glial fibrillary acidic protein (GFAP), a key component of astrocyte intermediate filaments. Accompanied by the transformation of the astrocyte, drastic changes in the morphology, gene expression, and executed functions occur depending on the context and region of the brain [5]. Additionally, reactive astrocytes actively participate in regulating inflammatory progression and resolution and neuronal functions by releasing a multitude of secretory mediators (such as tissue damage–associated molecules, cytokines, chemokines, and growth factors) that act as signaling molecules to interact with other cell types, including neurons, microglia, endothelial cells, and infiltrating leukocytes. Modulation of reactive astrocytes thus appears to be a viable approach for controlling the inflammatory response and improving outcomes in ischemic stroke. Although many molecules, involving interleukins, fibrinogen, STAT3, and NF-κB, promote astrocyte activation, few factors that directly act on astrocytes and suppress their activation have been identified. Therefore, elucidating the molecular signals involved in astrocyte activation and the potential factors that interfere with this activation is paramount.

Fibroblast growth factor 21 (FGF21) is a unique member of the FGF superfamily, that rarely exhibits mitogenic action and is likely the only FGF capable of acrossing the blood-brain barrier (BBB) because of its low affinity for heparin. Like other FGFs, FGF21 performs diverse actions by activating FGF receptors; however, efficient receptor engagement and signal transduction also require a specific coreceptor of the membrane-spanning protein β-klotho. The functions of FGF21 reportedly include regulating various biological and cellular responses, such as inflammatory modulation, energy metabolism, vascular homeostasis, oxidative stress, and tissue repair [6–8]. Recently, FGF21 has emerged as a promising target for treating CNS disorders. For example, FGF21 has been proposed as an early biomarker of dysregulated neuronal mitochondrial dynamics and endoplasmic reticulum (ER) stress conditions [9] that could act on oligodendrocyte precursor cells (OPCs) and promote remyelination in neurological disorders [10]. In a disease model of ischemic damage, FGF21 modulated NF-κB-dependent neuroinflammation in microglia/macrophages [11], and PI3K/Akt mediated neuronal apoptosis [12, 13] via its effect on FGF receptor 1(FGFR1) [14]. Moreover, the molecular mechanism underlying the role of FGF21 in maintaining BBB integrity has been reported to partially rely on endothelial-based therapy through the formation of an FGF21/FGFR1/β-klotho ternary complex and PPAR-γ activation [15, 16]. In addition to being distributed on the surface of neurons, microglia, and endothelial cells, FGFR1 is abundant on the surface of astrocytes [17]. However, studies on neurological diseases have paid little attention to the effects and detailed mechanisms of FGF21 in astrocytes, and the potential efficacy of FGF21 on astrocytes under normal and ischemic conditions is still unclear.

Our observation of markedly elevated serum levels of FGF21 in stroke patients and transient middle cerebral artery occlusion (tMCAO) mice established a rationale for further investigation of the role of FGF21 in the pathological progression of brain ischemia. In this study, we explored the role of FGF21 in regulating astrocyte responses under ischemic conditions via genetic and pharmacological approaches. Our findings reveal that FGF21 is a key modulator of astrocyte reactivity and participates in controlling the severity of neuroinflammation and brain injury after ischemia.

## Materials and methods

### Animals and drug administration

C57BL/6 mice weighing 20–25 g were obtained from the Animal Center of the Chinese Academy of Sciences (Shanghai, China). C57BL/6 Wild-type (WT) and FGF21 knockout (FGF21^−/−^, KO) mice were supplied by the Chinese-American Research Institute for Diabetic Complications (Wenzhou Medical University, China). Mice were housed in a temperature- and humidity-controlled, specific pathogen-free facility at the Medical University of Wenzhou with a 12-h light-dark cycle. Food and water are free to access. All animal experiments were authorized by the Laboratory Animal Ethics Committee of Wenzhou Medical University, and the procedures were conducted in compliance with the National Institutes of Health Guide for the Care and Use of Laboratory Animals (IACUC no: 2021-232).

Recombinant human FGF21 (rhFGF21) (Wenzhou Medical University, China) was dissolved in a sterile 0.9% normal saline solution. rhFGF21 (1.5 mg/kg) or vehicle control (sterile 0.9% normal saline solution, the volume of vehicle is the same as that of rhFGF21 solution) was injected intraperitoneally at the end of reperfusion and was administered at 12 h intervals for 7 d [11, 18].

### Human subjects

Blood samples were collected from stroke patients (excluding those with complications) and healthy subjects for FGF21 concentration measurements. Ischemic stroke patients with large artery atherosclerosis (thrombotic) were screened and included; these patients received routine lipid-regulating, antiplatelet and fluid rehydration treatments. On the 3rd day after admission, blood samples from fasting patients were collected via venipuncture. The study was carried out in compliance with the Declaration of Helsinki and approved by the Research Ethics Committee of Wenzhou Medical University (2021-K-73-02). Informed consent was obtained from all participants. Clinical information is available in Supporting Information Table S1.

### Transient middle cerebral artery occlusion

Mice were subjected to transient middle cerebral artery occlusion (tMCAO) by introducing the intraluminal filament technique as previously described [19]. In brief, each animal was anesthetized with 1.5% isoflurane in a mixture of 30% O_2_/68.5% N_2_O, and its rectal temperature was maintained at 36.5–37.5 °C. A midline skin incision was made at the neck and the left common carotid artery (CCA) was isolated and temporarily closed. Subsequently, the internal carotid artery (ICA) and external carotid artery (ECA) were exposed, and a silicone rubber-coated nylon monofilament (L1800, Jialing Company, Guangdong, China) was inserted into the ICA from the ECA. The occluding filament was left in the middle cerebral artery (MCA) for 60 min. A laser Doppler flowmeter (MedWrench, Brentwood, USA) was used to detect cerebral blood flow (CBF), and mice with CBF reductions of more than 80% compared with the baseline were selected. Sham-operated mice underwent identical anesthesia and surgical procedures without filament inserted into the MCA. The surgical MCAO procedure was performed on a total of 432 mice (including 330 C57BL/6 mice and 102 FGF21^−/−^ mice); the mortality rate was 8.5% for C57BL/6 mice (28 of 330) and 12.7% for FGF21^−/−^ mice (13 of 102). In addition, the rate of exclusion due to inadequate reperfusion, hemorrhagic transformation, or a modified Neurological Severity Score < 6 or > 13 were 12.4% for C57BL/6 mice (41 of 330) and 13.7% for FGF21^−/−^ mice (14 of 102).

### Enzyme-linked immunosorbent assay (ELISA)

Blood serum was prepared from fresh blood collected from stroke patients or mice via centrifugation at 1500 × *g* for 15 min, and the concentration of FGF21 was measured using a human/mouse FGF21 ELISA kit (EH188RB/EEL084, Invitrogen, California, USA) in accordance with the manufacturer’s instructions. The intraassay precision was evaluated in triplicate using six known quantitative standards.

### TTC staining

After euthanized, the brain tissue was carefully removed and cut into 1-mm-thick coronal slices on a mouse brain matrix slicer (WPI, Florida, USA). Subsequently, the slices were immersed with 2% 2,3,5-triphenyl tetrazolium chloride (TTC, Sigma, dissolved in phosphate-buffered saline) for 10 min, stained, and then stored in 4% paraformaldehyde overnight. The infarcted area was measured using ImageJ software (NIH Image, Bethesda, USA).

### Neuroimaging

Brain infarction in live mice was measured with a 9.4 T MRI instrument (BioSpec 94/20 USR, Bruker GmbH, Billerica, MA, USA). The experimental setup consisted of a 20 cm horizontal hole magnet, a 114 mm high-performance gradient magnetic field device (440 mT/m), an 86 mm transmitter coil and a mouse brain surface coil. The mice were anesthetized with inhaled 1.5% isoflurane through a face mask. During the scan, the breathing of the mice was monitored with an animal monitoring and control system with sensor pads deployed under the abdomen. T2-weighted images were obtained via rapid acquisition and fat-suppressed relaxation enhancement (RARE) sequences. The following scan parameters apply TR = 2000 ms. Effective TE = 33 ms. Rarity factor = 8. Obtain 20 slices with a thickness of 0.7 mm. Image size = 256 × 256; Field of view = 2000 × 2000.

### Neurobehavioral tests

Neurobehavioral assessments were conducted at 1, 3, 5, 7, 11, and 14 d after MCAO. All the mice received training for 3 consecutive days before undergoing neurobehavioral testing. Behavior data recorded before ischemia–reperfusion injury were used as the preoperative data. The modified neurological severity score (mNSS) was calculated, and the corner turning test, rotarod test, forelimb grip strength test, and adhesive removal test were performed to evaluate neurological deficits, sensorimotor abnormalities, motor balance and coordination, forelimb strength, and sensory ability.

#### MNSS

The mNSS scale was used to evaluate neurological deficits at days 1, 3, 5, 7, 11, and 14 after MCAO surgery. The mNSS items were graded on a scale of 0–14, and the scale was used to assess different neurological functions, including motor abilities (muscle strength and movement abnormalities), sensory capabilities (visual, tactile, and proprioceptive senses), and reflex responses (pinna, corneal, and startle reflexes), in the mice [20]. Points were assigned when the mice were unable to perform the tests or lacked the reflexes being assessed. Higher scores represented more severe damage.

#### Corner turning test

The corner test was used to access sensorimotor abnormalities as previously described [20]. A corner with an angle of 30° was made using two cardboard pieces, and the mice were allowed to enter the corner spontaneously. Normal mice turned either left or right, whereas MCAO/reperfusion-induced mice preferentially turned toward the side. The percentage of right turns over 20 trials was calculated.

#### Forelimb grip strength

The forelimb grip strength of the mice was tested with a recording grip meter according to previously reported methods [21]. In brief, the mice were held by the tail and allowed to grasp a wire ring connected to a strain gauge. The force required to pull the subject away from the wire was recorded as the grip strength.

#### Rotarod test

To assess motor balance and coordination, the rotarod test was performed using a rotarod apparatus (Comerio, Solbiate Olona, Italy) as previously described [22]. The mice were placed onto the rungs of the accelerating rotarod, and the speed was increased slowly from 5 to 40 rpm. The time that the mice remained on the rotarod was measured (within a maximum recording time of 5 min). The mean duration (in seconds) over three individual trials was recorded.

#### Adhesive removal tests

The adhesive removal test measured tactile responses to sensorimotor asymmetries and was performed as previously described [23]. A small adhesive dot was attached to the forepaw of each mouse, which was then placed in a cleaned cage in a calm mood. The amount of time (in seconds) required to contact and remove the tape from the forepaw was subsequently recorded (upper limit of 5 min). Before surgery, mice that exhibited no response or took a long time (≥20 s) to remove the dot in the training phase were excluded.

### Morris water maze test

Cognitive deficits after cerebral ischemia were determined via the previously described [24] Morris water maze test, which includes 2 parts: the cued test and the probe test. The cued test was used to assess spatial learning ability and was conducted at 21-25 d after MCAO, with 3 trials on each day. The time spent finding the hidden platform was recorded. On day 26, memory consolidation was assessed with the probe test, in which the hidden platform was removed. The number of times the mouse crossed the platform area and the time spent in the target quadrant where the platform was previously located were recorded.

### Immunohistochemistry and image analysis

Brains tissues were harvested and post-fixed in 4% PFA at 4 °C overnight. Subsequently, the brains were embedded in paraffin and sectioned into 5 μm slices. Followed by xylene deparaffination, ethanol rehydration, and incubation with 3% H_2_O_2_ (diluted in PBS solution) for 15 min. Then, the sections were boiled in citrate buffer for 2 min to retrieve antigen, and nonspecific antibody binding was blocked by 5% bovine serum albumin (BSA) for 30 min at 37 °C. Rabbit monoclonal anti-FGF21 (1:250, ab171941, Abcam) were incubated with tissue sections at 4 °C overnight, followed by incubation in goat anti-rabbit secondary antibody (1:800, ab6721, Abcam) for 2 h at 37 °C. Sections were colored with DAB kit (ZSGB-BIO, Beijing, China), and then counter-stained with hematoxylin (Beyotime Institute of Biotechnology, Shanghai, China). Finally, the resulting images were examined under white light using a Nikon ECLIPSE 80i microscope (Nikon, Tokyo, Japan) and analyzed using ImageJ software. The percent of ipsilateral FGF21-positive area increase was calculated using the following formula: [1-(area of ipsilateral FGF21 staining/area of contralateral FGF21 staining)] ×100%.

### Immunofluorescence staining

Brain sections were blocked with 5% BSA for 1 h at 37 °C, and incubated with mouse monoclonal anti-FGF21 (1:300, NBP2-67275, NOVUS), along with different markers including Rabbit monoclonal anti-NeuN (1:200, ab177487, Abcam), goat polyclonal anti-GFAP (1:200, ab53554, Abcam)/Mouse anti-GFAP (1:200, 3670S, CST), and goat polyclonal anti-Iba1 (1:500, ab48004, Abcam)/Rabbit anti-Iba1 antibodies (019–19741, Wako pure, Tokyo, Japan), BDNF (1:200, ab108319, Abcam), NGF (1:200, ab6199, Abcam) at 4 °C overnight. Then the sections were treated with Alexa Flour 488-conjugated donkey anti-mouse IgG (1:800, ab150105, Abcam), Alexa Flour 488-conjugated goat anti-Rabbit IgG (1:800, ab150077, Abcam), Alexa Flour 647-conjugated donkey anti-mouse IgG (1:800, ab150115, Abcam), Alexa Flour 647-conjugated donkey anti-goat IgG (1:800, ab150131, Abcam) or Alexa Flour 647-conjugated donkey anti-rabbit IgG (1:1000, ab150075, Abcam) for 1 h at 37 °C. After rinsing by PBST, Fluoroshield Mounting Medium with DAPI (ab104139, Abcam) was used to stain the cell nuclei. All fluorescence images were observed using a Leica confocal laser microscope (Leica, Wetzlar, Germany).

### Histological staining

Hematoxylin-eosin (HE) staining was performed to evaluate the histopathological changes in brains based on the previous protocol [25]. Brain sections were dried at 60–65 °C for 30 min, brain sections were subjected to H&E solution (Beyotime Institute of Biotechnology) after deparaffinized and rehydrated. Images were captured using a Nikon Eclipse 80i microscope (Nikon) and analyzed by ImageJ software. The residual volume was calculated using the following formula: (ipsilateral hemisphere volume /contralateral hemisphere volume) ×100%.

### Flow cytometry

Single-cell suspension was prepared from the brain, spleen, and blood as previously described [11, 20], and then stained with fluorochrome-conjugated antibodies. All antibodies tagged with one of the following fluorescent labels: allophycocyanin (APC), fluorescein isothiocyanate (FTIC), phycoerythrin (PE), PE-Cy7 or PerCP-Cy5.5, were purchased from BD Bioscience, eBioscience, or Miltenyi Biotech. The antibodies to mouse antigens were applied as following: CD86-PE (A17199A, 159203), CD68-PE (GL-1, 105008), CD206-PE (C068C2, 141706), F4/80-APC (BM8, 123116), CD11b-PerCP-Cy5.5 (M1/70, 101230), CD45-PE/Cy7 (30-F11, 103114), CD3-PerCP-Cy5.5 (17A2, 100217), CD8-FITC (53-5.8, 140404), CD4-APC (GK1.5, 100412), Ly6G-PE (1A8, 127608), Ly6C-APC (HK1.4, 128016), NK1.1-APC (PK136, 180710) (BioLegend, San Jose, USA), GFAP (GA5, 53-9892-82, eBioscience). Antibody staining was conducted according to their instructions, and single-cell analysis was performed by FACS Aria II flow cytometer (BD Biosciences, San Jose, USA). Gates were set by FMO controls and analyzed with FlowJo software (Version 10, Informer Technologies, USA).

### Fluorescence-activated cell sorting (FACS)

Microglia of the mouse brain tissue were isolated by FACS. After the single-cell of the ipsilesional or contralesional brain was prepared, microglia were stained with APC-conjugated CD45 (30-F1, 103112) and PE-conjugated CD11b (M1/70, 101208) antibody (BioLegend) for 30 min at 4 °C in dark. Unstained antibody was washed off by PBS and the population of microglia was collected by CD45^intermediate^CD11b^+^ gate setting on Aria II cell sorter.

### Magnetic-activated cell sorting (MACS)

Following a stroke in mice, astrocytes were isolated using magnetic-activated cell sorting with the Anti-ACSA-2 microbead kit (130097678, Miltenyi Biotec, Bergisch Gladbach, Germany). Brain mononuclear cells obtained from the contralesional or ipsilesional hemisphere were resuspended in 80 μL of 0.5% BSA solution in PBS and blocked with FcR reagent for 10 min. Astrocytes were labeled using anti-ACSA-2 microbeads through incubation, followed by washing off the unstained microbeads with BSA solution. The collected cells were resuspended in 1 mL BSA solution and loaded onto an LS column within the magnetic field of a MACS Separator. After washing, the magnetically labeled astrocytes were trapped on the column and then eluted into a collection tube post-column removal. The purity of astrocytes was confirmed to be above 90% by staining with ACSA-2 antibody (IH3-18A3, 130-117-535, MACS).

### Primary microglia and astrocyte cultures

Cortical microglia and astrocyte cultures were isolated and purified from 2-day-old neonatal Sprague–Dawley rats as reported previously [26]. In brief, after digesting and filtering, the mixed cells were plated on 75 cm^2^ flasks and cultured in DMEM/F12 medium containing 10% FBS and 1% Pen/Strep for 14 d. Mixed medium with a trypsin solution (0.25% trypsin diluted 1:4 in DMEM/F12) was induced for 30 min, microglia remained attached to the bottom of the well, while other cells were detached [27].

Mixed glial cultures were shaken at 220 rpm for 4 h, and at 180 rpm overnight to remove non-astrocytic cells. Astrocytes were dissected and reseeded on poly-*L*-lysine-coated plates. After the cells reached 80%–90% confluence, astrocytes were stimulated with LPS (200 ng/mL) and administered with rhFGF21(100 nM), PD173074 (10 μM) for 4 h. Gene assays of IL-1β and TNF-α in difference stimulated astrocytes were then performed by quantitative reverse transcription polymerase chain reaction (qRT-PCR).

To detect the action of rhFGF21-treated astrocytes on neurons after OGD/R, we first treated astrocytes with vehicle- or 100 nM of rhFGF21 (Wenzhou Medical University, China) for 24 h, and then the cultures were switched to neurobasal medium containing 2% B27, 0.5 mM *L*-glutamine and 1% Pen/Strep subsequently. Another 24 h later, an astrocytic conditioned medium was collected and transferred to neurons during the reoxygenation for further experiments.

### Primary cortical neuron cultures and oxygen glucose deprivation (OGD)/reoxygenation (R)

Primary cortical neurons were prepared from embryonal brains (16–18 d) of Sprague–Dawley rats as we previously described [12]. Briefly, the separated cortices were digested by 0.25% trypsin-EDTA (Invitrogen, California, USA), and were filtered by a cell strainer. Then the dissociated cortical cells were seeded at poly-*D*-lysine (Sigma-Aldrich) coated culture plates, and cultured in DMEM containing 5% FBS and 1% Pen/Strep at a density of 3 × 10^5^ cell/ml (3 ml for 6-well cell culture plate, 0.5 ml for 24-well cell culture plate). After 20 h of seeding, a neurobasal medium (Invitrogen) containing 2% B27 (Invitrogen), 0.5 mM *L*-glutamine (Sigma-Aldrich), and 1% Pen/Strep replaced the medium. The neurons cultured for 8–9 days after seeding were used for further analysis.

To simulate the ischemic conditions in vitro, we performed OGD/R experiments as previously reported [28]. The primary neurons cultured with serum-glucose-deprived mediums were transferred to an anaerobic chamber (95% nitrogen and 5% CO_2_) for 2 h. At the end of the OGD treatment, a conditioned medium from astrocytes was added, and the cultures were placed in a normal atmosphere for 24 h.

### Cell counting kit-8 (CCK-8) assay

Cellular viability was subjected to CCK-8 (Beyotime Institute of Biotechnology) assay. After the cells were treated, the CCK-8 solution was added to the culture medium (CCK-8 diluted 1:10 in medium), which was then incubated in the dark for 4 h at 37 °C. The optical density (*OD*) was measured at 450 nm (BioTek, Winooski, VT, USA). The percentage of cellular viability was calculated using the following equation: (*OD* value/control *OD* value) ×100%.

### Western blotting analysis

The brain tissues and cells were homogenized in RIPA lysis buffer (Sigma-Aldrich), 1% PMSF (Beyotime Institute of Biotechnology), and 1% phosphatase inhibitors (non-added in p-FGFR1 assay) (Solarbio, Beijing, China). A BCA protein assay kit was used to measure the protein concentrations, and the amount of protein (100 μg in vivo and 50 μg in vitro) was applied. Then, the membranes were followed by blocking with 5% nonfat milk for 2 h at room temperature and incubated with primary antibodies at 4 °C overnight. The primary antibodies used including rabbit monoclonal anti-FGF21 (1:1000, ab171941, Abcam), goat polyclonal anti-TGF-β (1:1000, ab92486, Abcam), rabbit polyclonal anti-PSD95 (1:1000, ab18258, Abcam), rabbit monoclonal anti-synaptophysin (1:20000, ab32127, Abcam), rabbit polyclonal anti-synaptotagmin1 (SYT1, 1:1000, ab131551, Abcam), rabbit monoclonal anti-BDNF (brain-derived neurotrophic factor, 1:1000, BS9896M, Bioworld), anti-NGF (nerve growth factor) antibody (1:1000, ab6199, Abcam), mouse monoclonal anti-VEGF (1:500, sc-7269, Santa Cruz), mouse monoclonal anti-IGF-1 (1:500, sc-518040, Santa Cruz), Occludin (1:1000, ab216327, Abcam), VE-cadherin (1:1000, ab33168, Abcam), anti-FGFR1 (1:500, ab10646), anti-p-FGFR1 (1:1000, ab59194), rabbit polyclonal anti-β-Actin (1:3000, AP0060, Bioworld), and rabbit monoclonal anti-GAPDH (1:3000, 5174, CST). On the second day, membranes were incubated with the appropriate secondary antibodies (goat anti-mouse, 1:10,000, BS12478, Bioworld; goat anti-rabbit, 1:30,000, ab6721, Abcam) for 1 h at room temperature. The bands were visualized with an ECL kit and then were detected using a ChemiDoc XRS+ Imaging System (Bio-Rad, California, USA). The gray values of the bands were analyzed using Image Lab 6.0 software (Bio-Rad).

### RNA isolation and real time-PCR analysis

Total RNA from primary astrocytes and brain tissues was isolated using Trizol reagent (Roche, Basel, Switzerland), and complementary DNA (cDNA) was obtained after reverse transcription (Prime Script RT Reagent Kit, Takara, Tokyo, Japan). Then Real time-PCR reactions were performed as follows: incubation at 50 °C for 2 min, denaturing at 95 °C for 10 min, followed by 40 cycles of 95 °C for 15 s, and 60 °C for 1 min. Primers were designed by Sangon Biotech (Shanghai, China), and the PCR primer sequence for each gene was provided in Table 1.

**Table 1:** Sequences of primers.

Primer sequence (5′-3′)	Product size (bp)	GenBank accession number
*Bdnf*		
Forward, GCGGCAGATCCCCCGACTGC	496	M61175
Reverse, AAGTTGTGCGCAAATGACTG		
*Igf-1*		
Forward, GACCTGCTGGCAATAGCTTC	468	NM021578
Reverse, GACTGGCGAGCCTTAGTTTG		
*Vegf*		
Forward, GTGACAAGCCAAGGCGGTGAG	113	NM_001287114.1
Reverse, GATGGTGGTGTGGTGGTGACATG		
*Tgf-β*		
Forward, GACCTGCTGGCAATAGCTTC	468	NM021578
Reverse, GACTGGCGAGCCTTAGTTTG		
*Clcf1*		
Forward, GTGTCATGGCAACTCTTGGCTACC	99	NM_207615.2
Reverse, TCTTCTGGAGGAAGTCGCTGTGG		
*Tgm1*		
Forward, GAGCCAGAGCCAGAGCCAGAG	96	XM_008770691.2
Reverse, GCAGGAGCAGCAGCCACAAC		
*Ptgs2*		
Forward, CGGACTGGATTCTACGGTGA	113	NM_017232.3
Reverse, CCCTTGAAGTGGGTCAGGAT		
*Cd14*		
Forward, CCCAAGCACACTCACTCAAC	106	XM_006254603.3
Reverse, ATCAGTCCTTTCTCGCCCAA		
*Cd109*		
Forward, GGCATGTTCCTGAACTCCTTCGC	119	NM_001108771.2
Reverse, CGCTCGGAATACTCTTGAGTGTCG		
*S100α10*		
Forward, ACCTGGACCAGTGCCGAGATG	128	NM_031114.1
Reverse, GCTCCAGTTGGCCTACTTCTTCTG		
*Gapdh*		
Forward, AGACAGCCGCATCTTCTTGT	323	X02231
Reverse, TACTCAGCACCAGCATCACC		
*Fgf21*		
Forward, CGACTGCTGCTGGCTGTCTTC	135	NM_020013.4
Reverse, GGCTTCAGTGTCTTGGTCGTCATC		
*β-actin*		
Forward, CACTGCAAACGGGGAAATGG	157	V01217
Reverse, TGAGATGGACTGTCGGATGG		

Total RNA extracted from sorted cells by miRNeasy Micro Kit (Qiagen, Germany) was reverse transcribed to cDNA by using Reverse Transcription kit (Qiagen) and amplified in step one on Bio-Rad CFX96 instrument System (Bio-Rad, Hercules, CA, USA) using Gene Assays with probes for *Cd68*(Mm03047343_m1); *Cd86*(Mm00444543_m1); *Cd206*(Mm01329359_m1); Tumor necrosis factor-α (*Tnf-α*, Mm00443258_m1); Interleukin-1β (*Il-1β*, Mm00434228_m1); Interleukin-6 (*Il-6*, Mm00443258_m1); Transforming growth factor beta (*TGF-β*, Mm01178820_m1); *Cxcl2*(Mm00436450_m1); *Cxcl1*(Mm04207460_m1); *Ccl3*(Mm00441259_g1); *VE-cadherin*(Mm00486938_m1); *Claudin-5*(Mm00727012_s1); Zo-1(Mm00493699_m1); *Occludin*(Mm00500912_m1); *Gfap*(Mm01253033_m1); *Lcn2*(Mm01324470_m1); *Vim*(Mm01333430_m1); *H2-T23*(Mm00439246_g1) ; Srgn(Mm01169070_m1); *H2-D1*(Mm04208017_m1); *Psmb8*(Mm00440207_m1); *Serping1*(Mm00437835_m1); *Clcf1*(Mm01236492_m1); *Tgm-1*(Mm00498375_m1); *Thbs1*(Mm00449032_m1); *Cd109*(Mm00462151_m1); *Ptgs-2*(Mm00478374_m1); *Cd14*(Mm00438094_g1); *S100a10*(Mm00501457_m1); *B3gnt5*(Mm00475226_m1); *Tm4sf1* (Mm00447009_m1); (Applied Biosystems, USA). PCR assays were performed on the Bio-Rad CFX96 System (Bio-Rad, Hercules, CA, USA) in accordance with the program described previously. Data of the target gene expression were normalized by β-actin, calculated by the 2^-ΔΔCT^ method, and expressed as the fold difference.

### Microvascular endothelial cell isolation

Brain microvascular endothelial cells were extracted, and the expression of the tight junction proteins claudin-5, ZO-1, VE-cadherin, and occludin was analyzed at both the protein and mRNA levels. The brain tissue from mice after MCAO was harvested and homogenized with a handheld homogenizer. Following centrifugation at 2000 × *g* for 10 min, the myelin in the supernatant was removed, leaving behind the collected deposits. Subsequently, 18% dextran was added, and the mixture was centrifuged at 4400 × *g*, and 4 °C for 15 min. The resulting supernatant contained a myelin layer, while the vascular pellets settled at the base of the tube. All steps were conducted at low temperatures, with the samples ultimately stored at −80 °C for further analysis.

### Evans blue (EB) extravasation

After 72 h of MCAO, BBB permeability was assessed by EB extravasation. A 2% EB saline solution was intraperitoneally administered and circulated for a duration of 4 h. After being deeply anesthetized, the mice were perfused transcardially with cold phosphate-buffered saline (PBS) to eliminate dye from the blood vessels. Tissue samples from the left hemisphere were promptly collected, weighed, and homogenized. After centrifugation at 1000 × *g* for 15 min, the supernatant was incubated with a TCA solution for 24 h, and the concentration of EB was determined via a spectrophotometer at an excitation wavelength of 610 nm.

### RNA sequencing

Two transcriptome libraries were constructed by total RNA samples from isolated astrocytes of FGF21^−/−^/WT mice after ischemia and vehicle-/rhFGF21-treated astrocytes. Briefly, total RNA was isolated using a Total RNA Extraction Kit, and a Qubit RNA Assay Kit and a Qubit 2.0 Fluorometer (Life Technologies, CA, USA) were used to measure the purity, concentration, and integrity of the RNA. Then, 0.1–1 μg of the total RNA was used as input material, and a VAHTSTM mRNA-seq V2 Library Prep Kit for Illumina® was used to generate sequencing libraries. PCR amplification and mRNA sequencing library construction were performed with fragmented mRNA. After the library quality was evaluated, mRNA sequencing was performed and sequencing reports were generated by Sangon Biotech (Shanghai, China).

### Statistical analysis

All value analysis was presented as the mean ± SD or SEM of at least three independent experiments and were performed using GraphPad Prism 9.0 (GraphPad Software Inc., San Diego, CA, USA). Two-way ANOVA with Sidak’s or Tukey’s multiple comparison was performed for multiple comparison. One-way ANOVA followed by Tukey’s tests when analyzing more than two groups. Unpaired Student’s *t*-test was used for the comparison of the two groups. A value of *P* < 0.05 was considered statistically significant.

## Results

### FGF21 levels are upregulated in the serum of stroke patients and the tMCAO mouse model

**Fig. 1:**
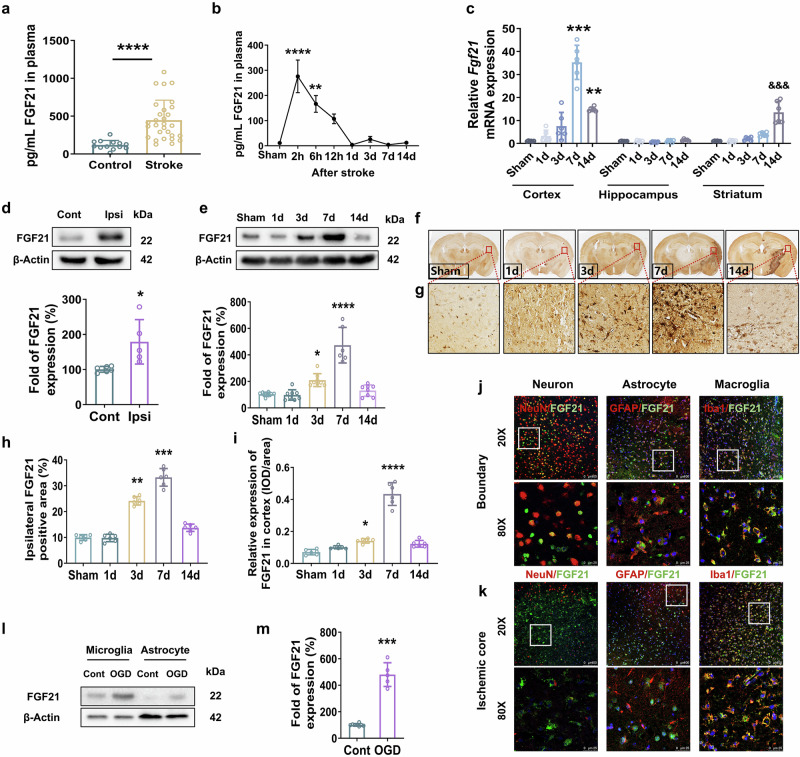
The expression of FGF21 was induced after cerebral ischemia. **a** The level of FGF21 in the serum of stroke patients was measured via ELISA. *n* = 13 for controls; *n* = 31 for stroke patients ^****^P < 0.0001 vs. control. **b** Quantification of serum FGF21 in mice during the acute and subacute stages after tMCAO via ELISA. *n* = 10 per group, ^**^P < 0.01, ^****^P < 0.0001 vs. the sham group. **c** FGF21 mRNA levels in ischemic **c**ortices, hippocampi, and striatum at 1, 3, 7, 14 d after transient MCAO/reperfusion. *n* = 6/group, ^**^P < 0.01, ^***^P < 0.001 vs. the sham group (Cortex), ^&&&^*P* < 0.001 vs. the sham group (Striatum). **d** Representative Western blot images and quantification of FGF21 in the ipsilesional (Ipsi) cortex compared with the contralesional (Cont) cortex at 7 d after MCAO/reperfusion. ^*^P < 0.05 vs. the Cont group; *n* = 6/group. **e** Representative Western blot images and quantification of FGF21 in the cortical penumbra at days 1, 3, 7, and 14 post-stroke. ^*^P < 0.05, ^****^P < 0.0001 vs. the sham group; *n* = 8 for sham, 1d-, 3d-, and 14d- group; *n* = 6 for 7d- group. **f**, **h** Representative images of immunohistochemical staining of FGF21 **f** and the percentage quantification of the injured ipsilateral FGF21-positive area **h**. **g**, **i** Magni**f**ied images of the cortical region **g** and the quantification of the average integrated optical density (IOD) of FGF21-positive staining **i**. *n* = 6/group. ^*^P < 0.05, ^**^P < 0.01, ^***^P < 0.001, and ^****^P < 0.0001 vs. the sham group; IOD/area: IOD per stained area. **j**, **k** Representative double-immunofluorescence staining images of FGF21 (green), neurons (red, NeuN-positive), astrocytes (red, GFAP-positive), and microglia (red, Iba1-positive) in the boundary zone **j** and the ischemic core **k** at 7 d after tMCAO. Scale bars = 100 µm or 25 µm. **l, m** Representative Western blot images and quantification of FGF21 in OGD/R-induced microglia and astrocytes. ^***^P < 0.001 vs. the Cont group; *n* = 5 per group. Data were expressed as mean ± SD, and statistical significance was determined by unpaired Student’s *t*-test or 1way-ANOVA with Tukey’s multiple comparison test.

FGF21 levels in the serum of stroke patients were substantially increased in the acute period after stroke onset compared with those in the control group (Fig. 1a). In the experimental tMCAO mouse model, FGF21 in the serum increased immediately after tMCAO, peaked at approximately 2 h, and returned to baseline by 1 d (Fig. 1b). We discovered that the mRNA levels of *Fgf21* in the ischemic brain were mainly concentrated in the cortical area at 7 d and 14 d after tMCAO, but that *Fgf21* levels in the striatum significantly increased at 14 d after tMCAO (Fig. 1c). Furthermore, compared with that in the contralesional cortex, the protein expression of FGF21 in the ipsilesional cortex was significantly increased (by 1.8-fold) at 7 d (Fig. 1d). In the cortical penumbras of tMCAO mice, FGF21 protein levels increased substantially from 1 d to 7 d before gradually declining to the levels found in the sham-operated mice over the next 7 d (Fig. 1e). Moreover, immunohistochemistry confirmed the increased FGF21 levels in the ipsilesional cortices (Fig. 1f, h) and cortical penumbras (Fig. 1g, i) at 3 d and 7 d after tMCAO. The tissues were subsequently stained for FGF21, and the cell sources of brain FGF21 at 7 d after tMCAO were assessed. FGF21-positive puncta were largely colocalized with Iba1-marked microglia, while some FGF21 was observed in neurons and astrocytes in the boundary zone (Fig. 1j). In the ischemic core, FGF21 was expressed mainly in microglia, and scarcely expressed in neurons and astrocytes (Fig. 1k). In in vitro tests, primary astrocytes and microglia were subjected to oxygen-glucose deprivation/reoxygenation (OGD/R), and FGF21 protein expression was detected after 30 min of OGD and 24 h of reoxygenation. FGF21 expression was extremely elevated (by 4.8-fold) in microglia and slightly induced in astrocytes after OGD/R (Fig. 1l, m). These results show that FGF21 is greatly induced by ischemia, and that microglia are more predisposed to produce FGF21 after ischemic insult.

### FGF21 deficiency aggravates brain infarction and neurological deficits

**Fig. 2:**
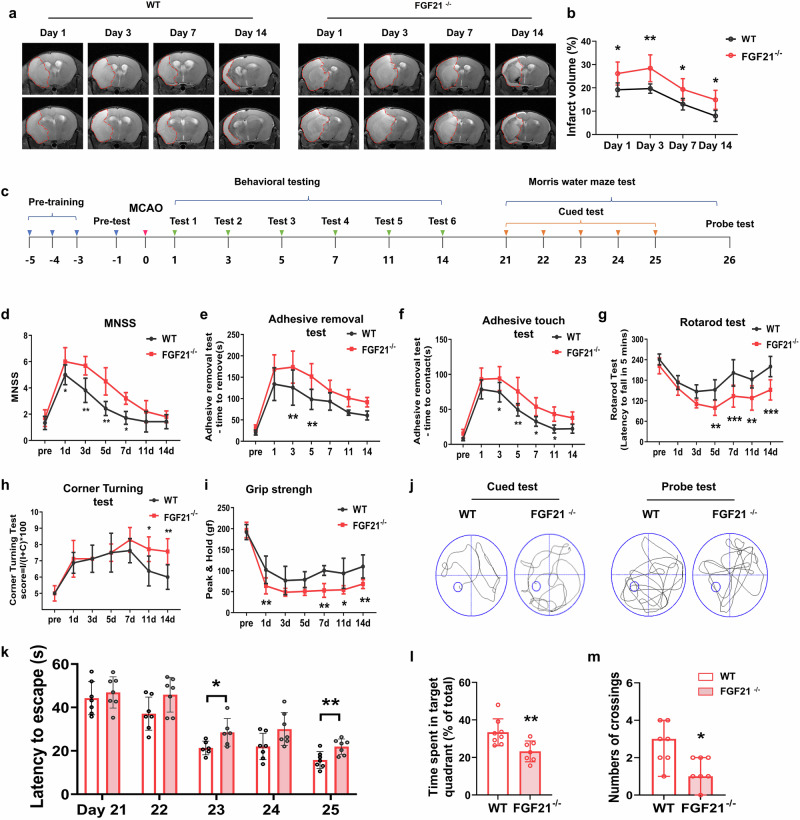
Exacerbated ischemic brain damage in FGF21^−/−^ mice. **a** The representative images of 9.4T-MRI show the time course of brain infarcts (outlined in red dashed line) in WT and FGF21^−/−^ mice. **b** Quantification of the infarct volume. *n* = 6/group. ^*^P < 0.05, ^**^P < 0.01 vs^.^ WT mice. **c** Schematic diagram of the timeline of neurological deficit assessments and cognitive testing. Neurological functions were evaluated with mNSS **d**, adhesive-removal test (time to contract **f**, and time to removal **e**), rotarod **g**, corner-turning **h**, and grip strength **i** tests at days 1, 3, 5, 7, 11, and 14 after surgery. *n* = 9 for the WT group, and *n* = 10 for the FGF21^−/−^ group; ^*^P < 0.05, ^**^P < 0.01, and ^***^P < 0.001 for FGF21^−/−^ group vs. WT group. **j** Spatial cognitive performances were determined by the Morris water maze test. Escape latency in the cued testing phase **k**, time spent in the target quadrant **l**, and numbers of platform crossing **m** in the probe test were measured. ^*^P < 0.05, ^**^P < 0.01 vs. the WT group. Results are presented as mean ± SD, and statistical significance was determined by unpaired Student’s *t*-test or 2way-ANOVA with Sidak’s multiple comparison test.

To evaluate the role of FGF21 in ischemic brain injury, we subjected FGF21^−/−^ and wild-type (WT) mice to focal cerebral ischemia and assessed the size of the brain infarction via triphenyltetrazolium chloride (TTC) staining and HE staining. Compared with WT mice, FGF21^−/−^ mice presented a larger infarct area at 3 d (Supporting information Fig. S1a) and 14 d (Supporting information Fig. S1b) after tMCAO. To track the variation in ischemic lesion size, T2-weighted imaging (T2-WI) was performed, and the results revealed that FGF21 deficiency aggravated ischemia-induced infarction growth from day 1 to day 3. A significant difference in infarct size was observed as early as 1 d after tMCAO and persisted to day 14 (Fig. 2a, b). A panel of neurobehavioral tests were subsequently performed (days 1, 3, 5, 7, 11, and 14) after tMCAO to test functional impairments after ischemic stroke, and spatial cognitive function was evaluated via the Morris water maze test at 21 d to 26 d after tMCAO (Fig. 2c). Compared with those in WT mice, sensory, motor, reflex, and balance functions in FGF21^−/−^ mice, as assessed by the modified neurological severity score (mNSS) test, significantly worsened at 1, 3, 5, and 7 d postinjury (Fig. 2d). Additionally, FGF21^−/−^ mice exhibited a marked deficit in sensorimotor recovery, as demonstrated by the delayed latency to touch and remove the adhesive tape at 3 and 5 d postinjury (Fig. 2e, f); a reduced duration in the rotarod test at 5, 7, 11, and 14 d postinjury (Fig. 2g); and an increased tendency to turn toward the contralateral side in the corner-turning test at 11 and 14 d postinjury (Fig. 2h). The grip strength test revealed that deficits in forelimb strength after stroke were exacerbated in FGF21^−/−^ mice at 1, 7, 11, and 14 d postinjury (Fig. 2i). No differences in sensorimotor function were observed between WT and FGF21^−/−^ mice in the pre-tMCAO test. Furthermore, in the Morris water maze test, compared with WT mice, FGF21^−/−^ mice presented a longer swimming latency in the cued test, indicating a significant decline in spatial learning ability after stroke (Fig. 2j, k). When the platform was removed, spatial memory capacity was more impaired in the FGF21^−/−^ mice than in the WT mice, as evidenced by the lower amount of time spent in the target quadrant and the reduced number of platform crossings in the probe test (Fig. 2l, m). Collectively, the behavioral assay data demonstrated that the absence of FGF21 led to persistent impairment of neurological function after ischemic injury.

### FGF21 deficiency promotes proinflammatory microglial activation after tMCAO

**Fig. 3:**
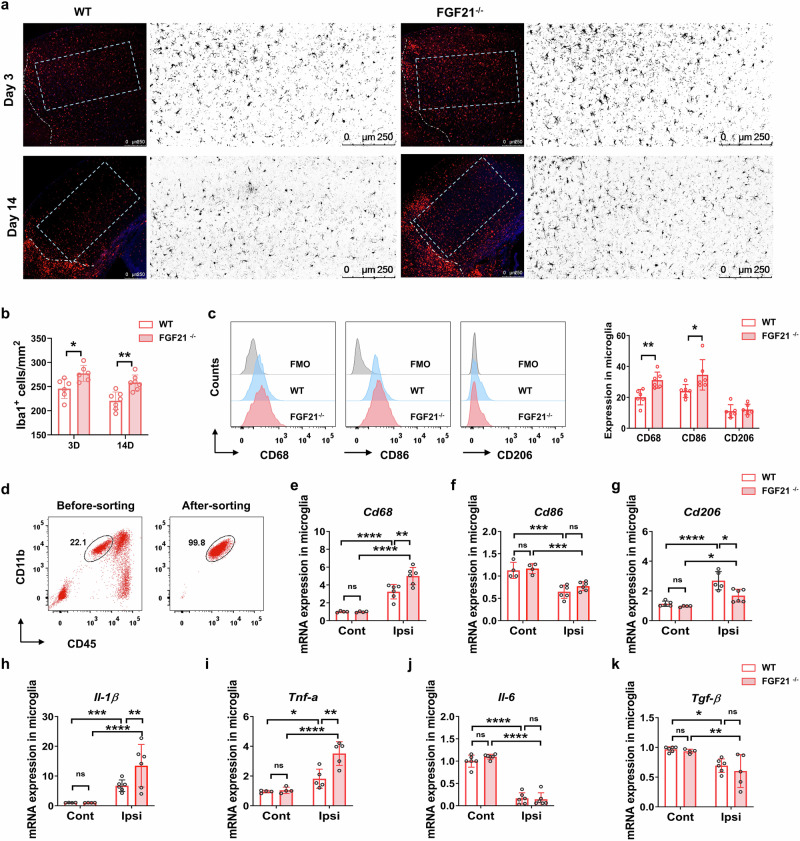
Altered microglial response and phenotype in FGF21^−/−^ mice after stroke. **a** Microphotographs and bar graph **b** of Iba1-positive (red) microglia in the perifocal cortex of WT and FGF21^−/−^ mice show the changes in microglia density at 3 and 14 d after tMCAO. *n* = 8/group. **c** FACS analysis and quantitative data of the expression of CD68, CD86, and CD206 in microglia (CD45^int^CD11b^+^) by FMO control. *n* = 6/group. ^*^P < 0.05, ^**^P < 0.01 by 2way-ANOVA with Sidak’s multiple comparison. **d** FACS images show the purity of microglia (> 99%) after cell sorting. **e**–**k** qRT‒PCR shows the mRNA expression of the microglial signature gene (*Cd68*, *Cd86*, and *Cd206*) and inflammatory cytokine (*Il-1β*, *Tnf-α*, *Il-6*, and *Tgf-β*) in the sorted microglia from the contralesional and ipsilesional brain of WT and FGF21^−/−^ mice. *n* = 6/group. ^*^P < 0.05, ^**^P < 0.01, ^***^P < 0.001, and ^****^P < 0.0001, determined by 2way-ANOVA with Tukey’s multiple comparison. Results are presented as mean ± SD.

On the basis of our previously published data concerning microglial modulation [11] in tMCAO mice that received intraperitoneal treatment with FGF21, we examined the microglia-mediated response to tMCAO injury in the context of FGF21 deficiency. Although the total numbers of resident microglia (CD11b^+^CD45^int^) in the ischemic hemisphere were similar between WT and FGF21^−/−^ mice (Fig. 5m), the density of Iba1^+^microglia in the perilesional cortex in FGF21^−/−^ mice was significantly greater than that in WT mice at 3 d and 14 d after tMCAO (Fig. 3a, b). Further analysis of microglial phenotypes revealed that the percentages of CD11b^+^CD45^int^CD68^+^ and CD11b^+^CD45^int^CD86^+^ proinflammatory microglia were increased in FGF21^−/−^ mice compared with WT control mice at 3 d after tMCAO, whereas the expression of CD206 was not altered by FGF21 deficiency (Fig. 3c). Moreover, we isolated contralesional and ipsilesional microglia (purity above 99%, Fig. 3d) from FGF21^−/−^ and WT mice at 3 d after tMCAO and assessed the mRNA expression of phenotypic genes (*Cd68*, *Cd86*, and *Cd206*) and inflammatory cytokines (*Il-1β*, *Tnf-α*, *Il-6*, and *Tgf-β*) via qRT-PCR. Compared with those in contralateral microglia, the mRNA levels of *Cd68* (WT: 3.41-fold vs. Cont; FGF21^−/−^: 4.96-fold vs. Cont; Fig. 3e), *Cd206* (WT: 2.69-fold vs. Cont; FGF21^−/−^: 1.78-fold vs. Cont; Fig. 3g), *Il-1β* (WT: 6.47-fold vs. Cont; FGF21^−/−^: 14.98-fold vs. Cont; Fig. 3h), and *Tnf-α* (WT: 1.87-fold vs. Cont; FGF21^−/−^: 3.67-fold vs. Cont; Fig. 3i) in microglia from the lesional brain parenchyma were dramatically increased in both WT and FGF21^−/−^ mice, but the levels of *Cd86* (WT: 0.58-fold vs. Cont; FGF21^−/−^: 0.66-fold vs. Cont; Fig. 3f), *Il-6* (WT: 0.16-fold vs. Cont; FGF21^−/−^: 0.13-fold vs. Cont; Fig. 3j) and *Tgf-β* (WT: 0.72-fold vs. Cont; FGF21^−/−^: 0.65-fold vs. Cont; Fig. 3k) were decreased. The transcript expression levels of these genes in the contralateral microglia did not differ between the WT and *FGF21*^−/−^ mice. In ipsilesional microglia after tMCAO, FGF21 deficiency increased the expression of proinflammatory cytokines (*Il-1β*: 2.28-fold vs. WT; *Tnf-α*: 1.93-fold vs. WT) and markers of the proinflammatory phenotype (*Cd68*: 1.54-fold vs. WT), but decreased the mRNA level of the anti-inflammatory marker *Cd206* (0.56-fold vs. WT). The mRNA expression of *Cd86*, *Il-6*, and *Tgf-β* in ipsilesional microglia did not differ between groups. Overall, FGF21 participates in regulating microglia-mediated neuroinflammation after stroke.

### FGF21 deficiency enhances astrocyte reactivity after stroke

**Fig. 4:**
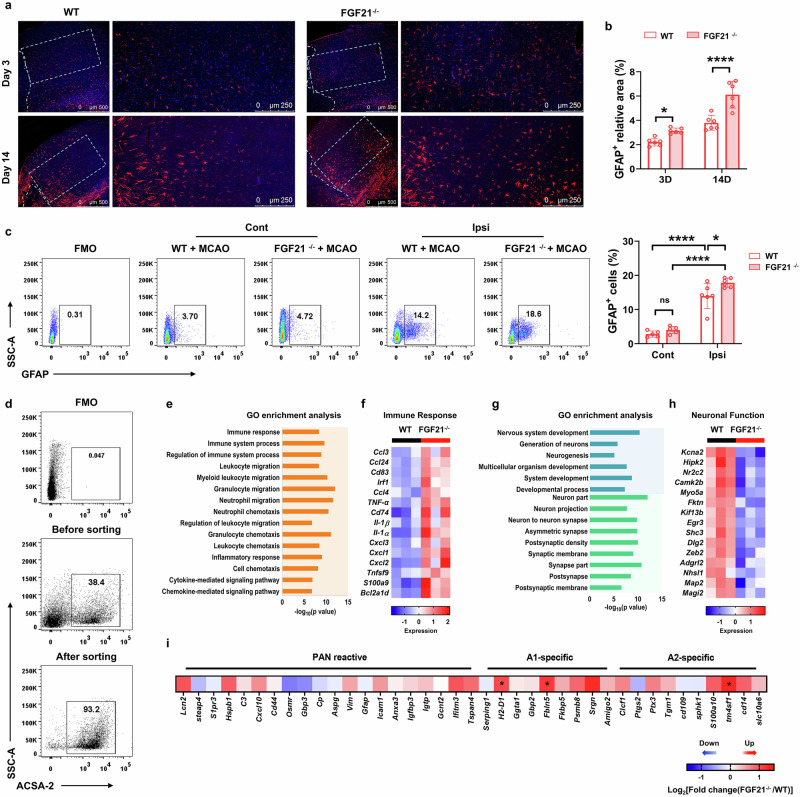
Increased astrocyte reactivity in FGF21^−/−^ mice after stroke. **a** Representative images of astrocytes immunostaining (GFAP, red; Hoechst, blue) in the perifocal cortex of WT and FGF21^−/−^ mice that were taken at days 3 and 14 after tMCAO. Scale bar=500 μm or 250 μm. **b** Quantitative assessment of the relative area of cells positive for GFAP in the indicated region of WT and FGF21^−/−^ mice. *n* = 6/group. ^*^P < 0.05, ^****^P < 0.0001, determined by 2way-ANOVA with Sidak’s multiple comparison. **c** Representative FACS plots and gate strategy of GFAP^+^ astrocyte in the ipsilesional and contralesional hemisphere of FGF21^−/−^ and WT mice at 3 d after tMCAO. ^*^P < 0.05, ^****^P < 0.0001, determined by 2way-ANOVA with Tukey’s multiple comparison. Results are presented as mean ± SD. **d** Astrocytes from the brain were isolated by MACS, and the purity of was verified by FACS analysis. **e**, **g** Representative Gene Ontology (GO) enrichment analysis of upregulated **e** and downregulated **g** genes expressed in astrocytes obtained from the ipsilesional brain of FGF21^−/−^ mice versus WT mice. **f**, **h** Heatmap showing the upregulated genes categorized in immune response **f**, and downregulated genes correlated with neuronal functions **h**. **i** RNA-sequencing data revealed the expression of PAN-reactive, A1- and A2-specific genes in the sorted astrocytes in the ipsilesional hemisphere of WT and FGF21^−/−^ mice at 3 d after tMCAO. *q* Value < 0.05, and Fold change > 2, for FGF21^−/−^ vs. WT group.

We subsequently assessed the activation status of astrocytes via immunostaining with GFAP and found that the GFAP-positive area in the perilesional cortex was larger in the FGF21^−/−^ mice than in the WT mice at 3 and 14 d after tMCAO (Fig. 4a, b). Flow cytometric analysis of GFAP also revealed that the number of GFAP^+^ astrocytes was apparently greater in the infarcted hemisphere than in the contralesional hemisphere in both WT and FGF21^−/−^ mice at 3 d after tMCAO; compared with WT mice, FGF21^−/−^ mice exhibited a dramatic increase in GFAP expression in the ipsilesional hemisphere (Fig. 4c).

**Fig. 5:**
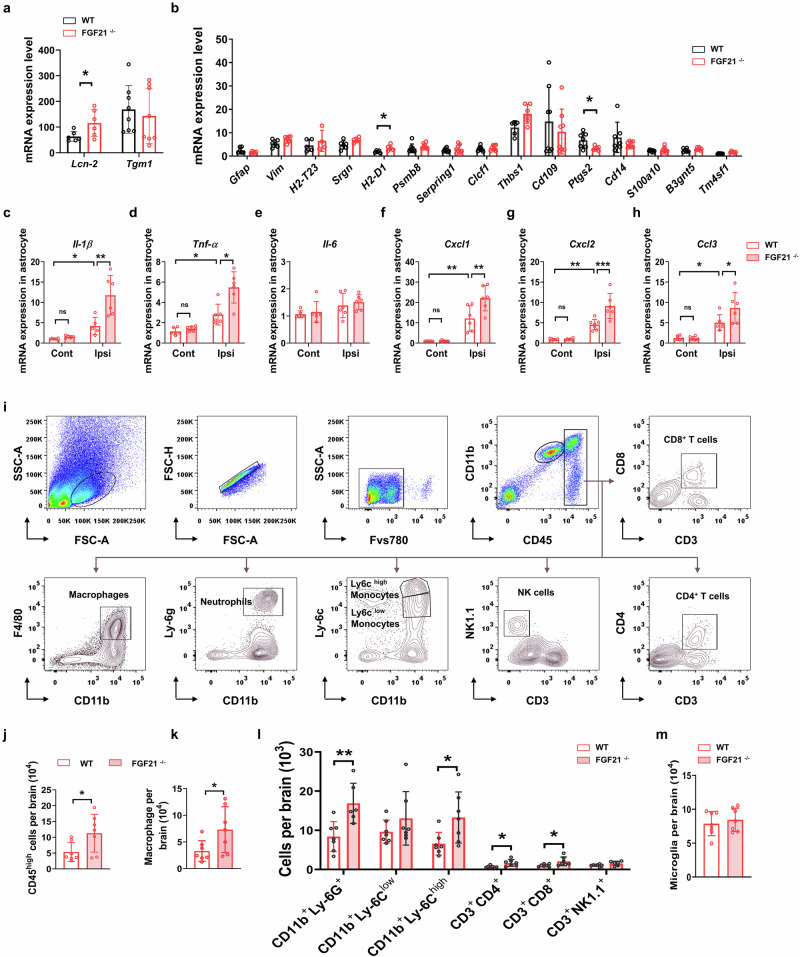
Increased astrocytic cytokine/chemokine expression and accumulation of leukocytes in FGF21^−/−^ mice. **a**, **b** Fold change of PAN-reactive (*Gfap*, *Vim*, and *Lcn-2*), A1- specific (*H2-T23*, *Srgn*, *H2-D1*, *Psmb8*, and *Serping-1*) and A2-specific (*Clcf-1*, *Tgm-1*, *Thbs-1*, *Cd109*, *Ptgs2*, *Cd14*, *S100a10*, *B3gnt5*, and *Tm4sf1*) genes in astrocytes in the ipsilesional hemisphere relative to the contralesional expression. *n* = 8/group. **c**–**h** Quantitative RT-PCR showed the mRNA expression level of inflammatory *Il-1β* **c**, *Tnf-а* **d**, *Il-6* **e**, *Cxcl1* **f**, *Cxcl2* **g**, and *Ccl3* **h**. *n* = 6/group. ^*^P < 0.05, ^**^P < 0.01, ^***^P < 0.001 by 2way-ANOVA with Tukey’s multiple comparison. **i** Flow cytometry plots show the gating strategy of microglia and infiltrating immune cell populations in the brain, including macrophages (CD45^high^CD11b^+^F4/80^+^), neutrophils (CD45^high^CD11b^+^Ly6G^+^), Ly6c^low^/Ly6c^high^ monocytes (CD45^high^CD11b^+^Ly6C^low^ and CD45^high^CD11b^+^ Ly6C^high^), NK cells (CD45^high^CD3^-^NK1.1^+^) CD8^+^T cells (CD45^high^CD3^+^CD8^+^)^,^ and CD4^+^T cells (CD45^high^CD3^+^CD4^+^). **j**–**m** Cell counts of brain-infiltrating leukocytes **j**, macrophages **k**, indicated immune cell subsets **l**, and brain-resident microglia **m** in the brain at 3 d after tMCAO. *n* = 8/group. ^*^P < 0.05, ^**^P < 0.01, determined by 2way-ANOVA with Tukey’s multiple comparison. Results are presented as mean ± SD.

To further explore the mechanism underlying regulation of the astrocytic response to ischemic damage by FGF21, we isolated ipsilesional GLAST^+^ (glutamate/aspartate transporter-positive) astrocytes (purity > 90%, Fig. 4d) from FGF21^−/−^ and WT mice at 3 d post-MCAO and performed a bulk RNA sequencing (RNA-Seq) analysis to identify the gene expression profiles of the purified astrocytes. A total of 155 upregulated genes (Supplementary data 1) and 156 downregulated genes (Supplementary data 2, q Value < 0.05, and Fold change > 2) were identified in the FGF21 knockout group compared with the WT group. Gene Ontology (GO) enrichment analysis revealed that in the FGF21^−/−^ group, upregulated genes were involved in immune and inflammatory responses (Fig. 4e, f and Supplementary data 3), whereas downregulated genes were involved in neuronal function (Fig. 4g, h and Supplementary data 4), suggesting that FGF21 deficiency could increase the degree of damage to neuronal function and is associated with inflammatory regulation. Specifically, the analysis of individual upregulated genes in the astrocytes of FGF21^−/−^ mice revealed changes related to cell migration and cell chemotaxis, such as *Tnf-а*, *Il-1а*, *Il-1β*, *Ccl3*, *Ccl24*, *Cxcl1*, *Cxcl2* and *Cxcl3* (Fig. 4f). However, the expression of genes clustered in “pan-reactive”, “A1-specific” and “A2-specific” motifs was not significantly influenced by FGF21 knockdown in astrocytes after ischemia, except for *H2-D1*, *Fbln5*, and *Tm4sf1*, which were upregulated in the FGF21^−/−^ group (Fig. 4i). Similarly, qRT‒PCR analysis of these astrocyte-specific genes revealed that FGF21 deficiency increased the expression of *Lcn-2* and *H2-D1*, and decreased the expression of *Ptgs2* (Fig. 5a, b).

### FGF21 deficiency increases the astrocyte-mediated inflammatory response and leukocyte infiltration

Next, the altered genes (*Il-1β*, *Tnf-а*, *Ccl3*, *Cxcl1*, and *Cxcl2*) revealed by the RNA sequencing data were verified via qRT-PCR. As anticipated, the expression of astrocytic *Il-1β* (WT: 3.90-fold vs. Cont; FGF21^−/−^: 9.65-fold vs. Cont; Fig. 5c), *Tnf-а* (WT: 2.60-fold vs. Cont; FGF21^−/−^: 4.41-fold vs. Cont; Fig. 5d), *Cxcl1* (WT: 11.08-fold vs. Cont; FGF21^−/−^: 18.16-fold vs. Cont; Fig. 5f), *Cxcl2* (WT: 5.12-fold vs. Cont; FGF21^−/−^: 10.11-fold vs. Cont; Fig. 5g) and *Ccl3* (WT: 4.13-fold vs. Cont; FGF21^−/−^: 7.42-fold vs. Cont; Fig. 5h) was dramatically greater in the ipsilesional hemisphere than in the contralesional hemisphere in both WT and FGF21^−/−^ mice. Compared with those in WT mice, the mRNA levels of these cytokines/chemokines were increased in the ipsilesional hemisphere of FGF21 knockout mice but not in the contralateral uninjured hemisphere. No significant difference in the transcript level of astrocytic *Il-6* was observed between the groups (Fig. 5e).

The upregulated genes related to leukocyte and granulocyte migration were enriched in the astrocytes of the FGF21^−/−^ mice compared with those of the WT mice; therefore, we investigated whether FGF21 deficiency influenced the brain’s cellular infiltrates after ischemia via flow cytometry (gating strategy showed in Fig. 5i). At 3 d after tMCAO, the number of infiltrating leukocytes (CD45^high^) was substantially greater in the ischemic brain parenchyma of FGF21^−/−^ mice than in that of WT mice (Fig. 5j). Among the CD45^+^ leukocytes, significantly increased cerebral accumulation of macrophages (CD11b^+^CD45^high^F4/80^+^), neutrophils (CD11b^+^CD45^high^Ly-6G^+^), Ly-6C^high^ monocytes (CD11b^+^CD45^high^Ly-6C^high^), and CD4^+^ (CD45^high^CD3^+^CD4^+^) and CD8^+^ T cells (CD45^high^CD3^+^CD8^+^) was also observed in the FGF21^−/−^ mice compared with the WT mice after tMCAO, whereas the numbers of Ly-6C^low^ monocytes (CD11b^+^CD45^high^Ly-6C^low^) and natural killer (NK) cells (CD45^high^CD3^-^NK1.1^+^) were similar between the two groups (Fig. 5k, l). Taken together, these data show that FGF21 deficiency leads to increased leukocyte infiltration after ischemia.

Moreover, we examined the peripheral immune response to FGF21 deletion under physiological and pathological conditions. Notably, uninjured FGF21^−/−^ mice exhibited a significantly increased percentage of CD4^+^ T cells in the spleen; and in the blood, the percentage of CD4^+^ T cells was increased in both normal and ischemic conditions. The number of CD8^+^ T cells in the blood of FGF21^−/−^ mice was greater than that in the blood of their WT littermates. In contrast, the cell compositions of Ly-6C^low^ and Ly-6C^high^ monocytes (CD11b^+^Ly6G^-^Ly6C^low^/CD11b^+^Ly6G^-^Ly6C^high^), neutrophils (CD11b^+^Ly6G^+^), NK cells (CD3^-^NK1.1^+^) and macrophages (CD11b^+^F4/80^+^) in the spleen and blood did not significantly differ between WT and FGF21^−/−^ mice (Supporting information Fig. S2a, c, e). These findings indicate that FGF21 deficiency leads to T-cell disturbances in the peripheral system. Furthermore, under ischemic conditions, FGF21 regulated macrophage polarization, as demonstrated by the increased expression levels of CD68^+^ and CD86^+^ macrophages in the blood of FGF21^−/−^ mice relative to their WT littermates. (Supporting information Fig. S2b, d, f).

To test whether increased leukocyte infiltration was driven by damage to the BBB, we measured the expression of tight junction-related proteins in isolated cerebral microvascular fragments at the protein and mRNA levels. Although there was no significant difference in the mRNA levels of *Claudin-5*, *VE-cadherin*, *Zo-1*, and *Occludin* between WT and FGF21^−/−^ mice in either the contralesional or ipsilesional microvascular fragments (Supporting information Fig. S3a), FGF21 deficiency resulted in decreased expression of *Occludin* and *VE-cadherin* at the protein level in the ipsilesional brain microvascular fragments (Supporting information Fig. S3b, c).

### rhFGF21 treatment suppresses the astrocyte-mediated inflammatory response and increases the expression of BDNF and NGF in astrocytes

**Fig. 6:**
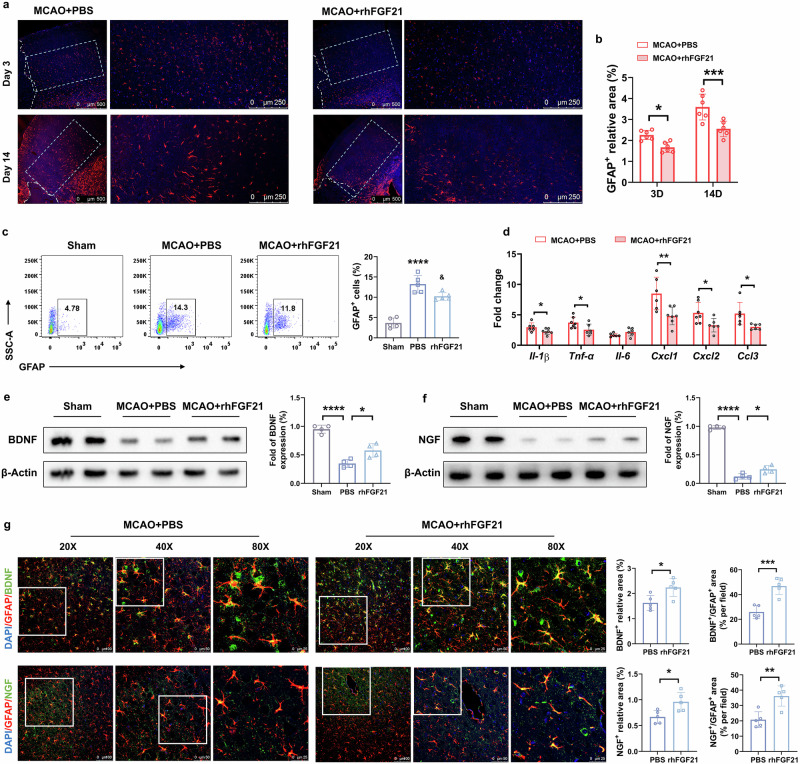
Altered astrocyte response in rhFGF21-treated mice after stroke. **a** Representative images of GFAP immunostaining in the perilesional brain tissue sections from tMCAO mice receiving PBS or rhFGF21, Scale bar=500 μm or 250 μm. **b** Relative GFAP-positive area in the cortical region surrounding the border of the lesion for 3 and 14 d post-injury. *n* = 6/group. ^*^P < 0.05, ^***^P < 0.001, by 2way-ANOVA with Sidak’s multiple comparison. **c** Gating strategy and quantification for GFAP^+^ astrocytes in the ipsilateral hemisphere 3 d after tMCAO. *n* = 6/group, ^****^P < 0.0001 vs. sham group ^&^*P* < 0.05 vs. MCAO group (1way-ANOVA with Tukey’s multiple comparison). **d** Quantitative RT-PCR for mRNA expression of *Il-1β*, *Tnf-α*, *Il-6*, *Ccl3*, *Cxcl1*, and *Cxcl2* in sorted astrocytes. *n* = 8/group. ^*^P < 0.05, ^**^P < 0.01, by unpaired Student’s *t*-test. **e**, **f** The expression levels of BDNF and NGF in the cortex around the infarcted zone were detected by Western blot, and normalized to β-actin. *n* = 4/group. ^*^P < 0.05, ^****^P < 0.0001, determined by 1way-ANOVA with Tukey’s multiple comparison**. g** Double-immunofluorescence staining for GFAP/BDNF and GFAP/NGF in the perifocal cortex under PBS or rhFGF21 treatment condition at 3 d after tMCAO. Scale bar=100, 50, or 25 μm. *n* = 5/group. ^*^P < 0.05, ^**^P < 0.01, ^***^P < 0.001, determined by unpaired Student’s *t*-test. Results are presented as mean ± SD.

To assess the effects of pharmacological modulation of FGF21 on the astrocyte response after stroke, we subjected MCAO mice to intraperitoneal treatment with rhFGF21 at 12 h intervals starting after reperfusion and measured GFAP expression in the cortical areas adjacent to the infarcted region. At both 3 and 14 d after the induction of ischemic injury, the relative GFAP-positive area in the peri-infarct cortex was lower in rhFGF21-treated mice than in PBS-treated mice (Fig. 6a, b). Flow cytometry analysis of GFAP revealed that rhFGF21 treatment markedly decreased the increased GFAP expression in the injured brain at 3 d after tMCAO (Fig. 6c). As mentioned above, FGF21 is potentially involved in modulating astrocyte-mediated inflammation and cell migration after ischemia. We next investigated the mRNA expression of the inflammatory genes *Il-1β*, *Tnf-а*, and *Il-6*, as well as the chemotactic genes *Ccl3*, *Cxcl1*, and *Cxcl2*, in sorted astrocytes. In response to rhFGF21 administration, MCAO-induced expression of *Il-1β*, *Tnf-а*, *Ccl3*, *Cxcl1*, and *Cxcl2* in astrocytes was suppressed (Fig. 6d). For in vitro experiments, we stimulated primary astrocytes with lipopolysaccharide (LPS) (200 ng/mL) to induce the expression of *Tnf-а* and *Il-1β*, which was reduced by rhFGF21 treatment. This mediation by rhFGF21 was abolished by the administration of PD173074 (a selective inhibiter of FGFR1) (Supporting information Fig. S4a, b).

Moreover, we found that rhFGF21 treatment markedly increased the production of the neurotrophic factors BDNF and NGF in the perifocal cortex at 3 d after tMCAO (Fig. 6e, f), particularly in astrocytes (Fig. 6g). Compared with those in WT mice, astrocytic BDNF and NGF expression in FGF21^−/−^ mice was decreased (Supporting information Fig. S5a, b, c).

### rhFGF21-treated astrocytes contribute to neuronal survival and upregulate synaptic protein expression

**Fig. 7:**
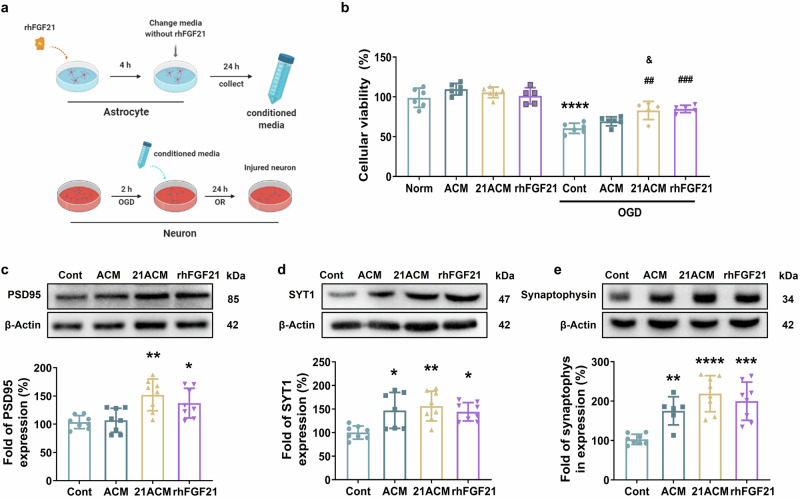
Effects of rhFGF21-stimulated astrocytes on neuronal survival and neuroplasticity after OGD/R exposure. **a** A flow chart of the experiment with conditioned medium transfer from astrocytes to neurons in culture. **b** Neuronal viability after conditioned medium treatment under normal or OGD/R conditions. ^****^P < 0.0001 vs. the Norm group; ^##^*P* < 0.05 and ^###^*P* < 0.001 vs. the Cont group; ^&^*P* < 0.05 vs. the OGD/R-ACM group (one-way ANOVA with Tukey’s multiple comparison); *n* = 6. **c**–**e** Representative Western blot images and quantification of PSD95, SYT1, and synaptophysin levels in OGD/R-induced neurons cultured with conditioned medium. Norm, normal, nontreated neurons; ACM, conditioned medium from normal astrocytes; 21ACM, conditioned medium from rhFGF21-treated astrocytes; rhFGF21, rhFGF21 treatment alone. *n* = 8. ^*^P < 0.05, ^**^P < 0.01, ^***^P < 0.001, ^****^P < 0.0001 and vs. the Cont group (one-way ANOVA with Tukey’s multiple comparison).

To ascertain whether FGF21 promotes beneficial neuronal outcomes by modulating astrocytic activation/reactivity, we treated astrocytes with rhFGF21 (100 nM), and transferred the conditioned medium to OGD-induced neurons (Fig. 7a). Of note, these media were protective; in particular, the conditioned medium from rhFGF21-treated astrocytes could protect neurons against OGD, as manifested by the results in CCK-8. Normal neuronal viability gradually decreased by 40% after 2 h of OGD and 24 h of reoxygenation; however, rhFGF21-treated astrocyte-conditioned medium (21ACM) effectively increased neuronal viability. rhFGF21 alone also exhibited a protective effect against OGD-induced neuronal death (Fig. 7b). Additionally, the protein level of postsynaptic density protein-95 (PSD-95), SYT1, and synaptophysin, which related to synaptic plasticity and neuronal function [29], were significantly promoted in the 21ACM- and rhFGF21-treated neurons compared with control neurons after OGD/R onset (Fig. 7c–e). Moreover, upregulated levels of SYT1 and synaptophysin were also present in the ACM-treat group compared to control (Fig. 7d, e). All these results proved the beneficial effects of rhFGF21-treated astrocytes on neuronal survival and neuroplasticity.

### rhFGF21 modulates the astrocytic reactivity and upregulates neurotrophic factors in vitro

**Fig. 8:**
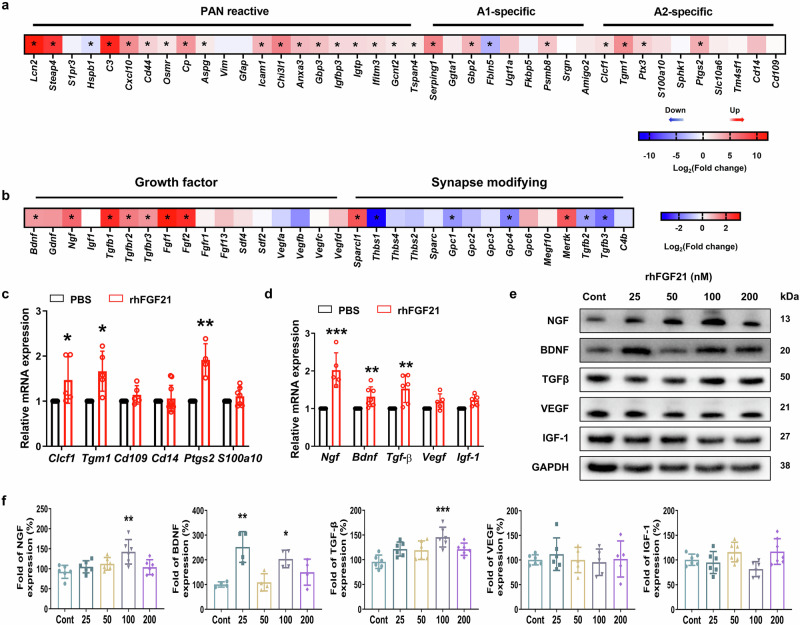
rhFGF21 alters astrocyte reactivity and induces the expression of trophic factors. **a** Analysis of RNA-seq data for astrocytes following treatment with FGF21 depicts the changes of genes in the PAN-reactive, A1- and A2-specific categories. (^*^P < 0.05, compared to non-reactive astrocytes). **b** Heatmap of growth factor and synapse-modifying genes expression changes between non-treated and FGF21-treated astrocytes. **c** Quantitative PCR for A2-specific gene after rhFGF21 treatment for 4 h. **d** Fold change of genes *Bdnf*, *Ngf*, *Vegf*, *Tgf-β*, and *Igf-1* in FGF21-treated astrocytes compared to Cont. *n* = 6. ^*^P < 0.05, ^**^P < 0.01 and ^***^P < 0.001 (unpaired Student’s *t*-test). **e**, **f** Representative Western blot images and quantification of NGF, BDNF, TGF-β, VEGF, and IGF-1 in cell lysates after rhFGF21 treatment for 24 h. *n* = 6. ^*^P < 0.05, ^**^P < 0.01, and ^***^P < 0.001 vs. the Cont group (one-way ANOVA with Tukey’s test).

To elucidate the potential impact of FGF21 on astrocytes, we treated astrocytes with rhFGF21 for 24 h (the morphology and cellular viability did not change, Supporting information Fig. S6a, b), and analyzed transcriptome differences between FGF21- and non-treated astrocytes by RNA-Sequencing. A total of 2380 differentially expressed genes (DEGs) were detected by gene sequencing bioinformatic analyses, of which 1350 genes were upregulated and 1030 genes were downregulated in the rhFGF21 group, that delineated in a volcano plot (Supporting information Fig. S6c). Especially, we profiled 40 genes that spanned 3 categorical bins related to astrocyte reactive profiles. Our findings indicate that rhFGF21-treated astrocytes were not particularly constrained to either “A1-specific” or “A2-specific” categorical cluster, with massive transcriptional changes predominantly present in the “Pan-reactive” motifs (Fig. 8a). Notably, rhFGF21 treatment significantly induced the gene expression of growth factors including *Bdnf*, *Ngf*, *Tgf-β*, *Fgf1*, and *Fgf2*, that have an active role in neurotrophy (Fig. 8b). To validate these results in gene sequencing, we examine the gene expression of partially DEG categorized in “A2” bin (*Clcf1*, *Tgm-1*, *Cd109*, *Cd14*, *S100a10*, and *Ptgs2*) or function on nerve growth (*Bdnf*, *Ngf*, *Tgf-β*, vascular endothelial growth factor (*Vegf*), and *Igf-1*) by qRT-PCR. Consistently, rhFGF21-treated astrocytes had remarkably increased gene expression of A2-specific *Clcf1*, *Tgm-1*, and *Ptgs2* and neurotrophic *Bdnf*, *Ngf*, and *Tgf-β* (Fig. 8c, d). Moreover, protein levels of BDNF, NGF, TGF-β, VEGF, and IGF-1 were also measured by Western blot and the results show that BDNF, NGF, and TGF-β were upregulated after treatment with rhFGF21 for 24 h, while VEGF and IGF-1 were not altered (Fig. 8e, f). These data revealed that rhFGF21 guided astrocytes into potentially beneficial phenotypes that are expected to accelerate neurobehavioral recovery by producing neurotrophic factors.

In the surfaces of astrocytes, we observed substantial expression of FGFR1 (Supporting information Fig. S6d). After the treatment of rhFGF21, the phosphorylation level of FGFR1 was significantly induced, and then abrogated by co-administration with PD173074 (a selective inhibiter of FGFR1), which suggests that rhFGF21 acts on astrocytes through the activation of FGFR1 (Supporting information Fig. S6e, f).

## Discussion

The present study revealed that FGF21 levels were increased in the serum of stroke patients as well as in an experimental tMCAO mouse model and that glial cells such as microglia and astrocytes are another source of cerebral FGF21 after stroke in addition to neurons. FGF21 deficiency leads to neurological deterioration after stroke, which is partly attributed to excessive immune responses that are amplified from astrocytes to recruited leukocytes. Following pharmacological intervention, anti-inflammatory responses and neurotrophic effects achieved by the action of FGF21 on astrocytes through FGFR1 were identified. Thus, FGF21 is a critical growth factor that modulates astrocyte reactivity and may be a potential therapeutic target for treating ischemic stroke.

In clinical practice, we found that endogenous FGF21 was robustly upregulated in the serum of stroke patients and animal models. The recognition that increased serum FGF21 expression in patients with obesity/type 2 diabetes (T2D) compensates for pathologic metabolic changes [30, 31] led us to further dissect the role of FGF21 in the ischemic cascade after stroke. Compared with plasma FGF21, which peaks at 2 h poststroke in mice, cerebral FGF21 is largely induced at 7 d after brain ischemia in the cortical region around the lesion, and at 14 d in the striatum. Considering that factors upregulated in the acute period after brain ischemia commonly result in a detrimental outcome (such as IL-6, IL-15 [20]), we wondered whether increased cerebral FGF21 expression during the subacute and delay phases could play a positive role. Previous studies on the cellular expression of FGF21 in the CNS revealed that FGF21 can be induced in neurons by cotreatment with lithium and valproic acid (VPA) (mood stabilizers) [32]. Thus, we determined the cell sources of cerebral FGF21 in the pathological context of ischemia. Consistent with the findings of Zheng et al. [14], increased FGF21 expression is induced in neurons in the penumbra after ischemic insult. In addition to neurons, glial cells also serve as sources of FGF21, with microglia being more susceptible to ischemic insult than astrocytes. These data further support the theory of *Salminen* et al. [33] that endogenous FGF21 is released by different cell types according to the degree of brain damage in a time- and context-dependent manner.

In addition to being expressed in the CNS, FGF21 is widely expressed in peripheral tissue, predominantly in the liver and pancreas. While FGF21 can cross the BBB [34], the concentration of FGF21 in the cerebrospinal fluid of healthy individuals is typically 40% of that found in plasma [35]. However, in specific conditions, such as pathological scenarios involving vascular barrier disruption or in normal tissues with high vascular permeability, a greater amount of FGF21 may be able to penetrate the BBB and exert its effects on the CNS. Therefore, to eliminate the interference of peripheral FGF21, FGF21^−/−^ mice were used in this study [10]. As previous reported, fasted FGF21^−/−^ mice exhibit impaired activation of the hypothalamic–pituitary–adrenal (HPA) axis and blunted release of corticosterone, which could lead to hypoglycemia and defective hepatic gluconeogenesis [36]. However, MRI did not reveal obvious alterations in the brain anatomy or cerebral vasculature (Supporting information Fig. S7a, b) under normal conditions. Upon the induction of ischemia, we observed a poorer outcome of FGF21 deficiency in the experimental tMCAO mouse model with enlarged brain infarction and exacerbated degeneration of long-term sensorimotor performance, suggesting that FGF21 acts as a neuroprotective regulator in the control of neural injury after ischemia; this result is supported by the RNA sequencing data showing that the expression of neuronal function-associated genes in astrocytes after tMCAO was decreased by FGF21 knockdown. These findings provide additional evidence for the therapeutic effect of rhFGF21 on ischemic stroke [11, 37]. Moreover, consistent with aggravated brain injury, FGF21^−/−^ mice exhibited substantially greater memory impairments during the recovery phase in the Morris water maze test; these results corresponded to the improved cognitive function in response to exogenous supplementation with FGF21 observed in a rat model of hypoxic ischemia [12].

Neuroinflammation is considered a pivotal mechanism of neuronal injury, and the participation of microglia and astrocytes is indispensable in neuroinflammation progression. Microglia serve as the central controllers of immune responses in the brain and their activation can elicit the generation of a diverse array of inflammatory mediators, such as inducible nitric oxide synthase (iNOS) and cytokines, leading to the development of CNS inflammation [38]. In contrast to the anti-inflammatory properties of exogenously supplemented FGF21 through the modulation of microglia/macrophages [11, 39], microglia and peripheral macrophages in FGF21^−/−^ mice with ischemia showed elevated activation of the proinflammatory phenotype (M1) and production of proinflammatory cytokines. These alterations in microglia/macrophages may directly or indirectly affect the outcome of ischemic brain injury.

Astrocyte activation, or astrogliosis, is a characteristic feature of the astrocytic response to ischemia and contributes to the progression or resolution of inflammation. After damage is removed, activated astrocytes typically reestablish their nonreactive state, which plays a key role in maintaining the homeostasis of neural tissue. Sustained astrocyte activation could be deleterious to functional recovery because activated cells inhibit axonal and cellular regeneration [40]. In this study, we found that, similar to β-integrin [41] and Smoothened [42] (a mediator of SHH signaling), FGF21 acts as a suppressor that controls astrocyte activation after ischemic injury. However, this function mediated by FGF21 is seemingly distinct from that of other FGFs; for example, basic fibroblast growth factor (bFGF) has been found to increase in conjunction with FGFR1 in reactive astrocytes after focal brain injury [43, 44] and to induce astrocyte reactivity [45], astrogliosis and scar formation [46], which is partially based on its mitogenic and morphogenic functions [43]. But FGF21 exerts very few mitogenic effects. However, in another study, the addition of FGF2 to astrocyte cultures resulted in a significant decrease in GFAP expression [47], implicating FGF2 as an inhibitor of astrocyte activation. Moreover, FGF4 is believed to be required for astrocyte differentiation [48], and FGF9 has been demonstrated to promote adult NPC proliferation, but inhibits the differentiation of GFAP-expressing astrocytes [49]. These studies prompt a reconsideration of the role of FGFs and FGF signaling in astrocyte function, and Kang et al. [4] reported that although FGF signaling inhibits the activation of astrocytes under normal or injured conditions, it also plays a role in promoting proliferation.

The analysis of the genetic profile of astrocytes isolated from WT and FGF21^−/−^ mice after ischemia revealed an overexuberant inflammatory state in the FGF21^−/−^ group, with elevated expression of proinflammatory cytokines and chemokines. CCL3, CXCL1, and CXCL2 are among the most commonly elevated chemokines in the ischemic context and are highly expressed in astrocytes [20, 50]. CXCL1 and CXCL2 are critical chemoattractants that recruit periphery neutrophils and are essential for transendothelial migration, which is also associated with disruption of the BBB [51]. CCL3 (also known as MIP-1а) has been shown to trigger monocyte/macrophage and microglial recruitment both in vitro [52] and in vivo [53] and to attract neutrophils in a CCR1-dependent manner [54]. The excessive expression of these chemokines in astrocytes due to FGF21 deficiency suggests that FGF21 participates in the suppression of chemokine-mediated immune cell infiltration. In fact, FACS analysis revealed that FGF21 deficiency facilitated the recruitment of blood-derived leukocytes, specifically macrophages, neutrophils, Ly-6C^high^ monocytes, and CD4^+^ and CD8^+^ cells, after tMCAO. Additionally, the mitigation of the gene expression of astrocytic CCL3, CXCL1, and CXCL2 by exogenous supplementation with rhFGF21 might explain why rhFGF21 alleviated macrophage recruitment in previous studies [11].

Despite the increased cerebral accumulation of peripheral leukocytes, which can be partially explained by the increased chemotactic effects of CCL3, CXCL1, CXCL2, and other chemokines, we cannot exclude the influence of BBB breakdown and peripheral immune cell expression. We found that FGF21 deficiency results in aggravated degradation of tight junction proteins in the BBB after ischemia, which may drive the increased infiltration of leukocytes. The disruption of the BBB is largely attributed to increased inflammation or the secretion of inflammatory cytokines and chemokines, such as TNF-α, IL-1α, IL-1β, CCL3, CXCL1, and CXCL2. Moreover, flow cytometry analysis of the composition of immune cells in the peripheral spleen and blood revealed that FGF21 deficiency increased CD8^+^ T-cell expression under ischemic conditions and upregulated CD4^+^ T-cell levels under both ischemic and uninjured conditions, suggesting that FGF21 deletion leads to T-cell disturbances in the peripheral system, which contribute to the infiltration of CD8^+^ and CD4^+^ T cells. Although some data are conflicting, CD8^+^ and CD4^+^ T cells are considered detrimental to the injured brain [55, 56]. Unfortunately, the potential impact of FGF21 or FGF signaling on the functional status of CD8^+^ and CD4^+^ T cells was not addressed in this study, and further research is needed.

Considering that endogenous expression and in vivo administration of FGF21 following stroke could impact multiple cell types (such as microglia), meaning that any observed alterations in astrocytes may be an indirect result of changes in other cell types, exogenous rhFGF21 was directly added to stimulate primary astrocytes in in vitro experiments. Bulk RNA transcriptomics was performed in astrocyte cultures treated with PBS or rhFGF21; although a transcriptional alteration in GFAP expression was not detected, dramatic changes in genes enriched in the “pan-reactive” category and some genes in the “A1-specific” and “A2-specific” modules were unveiled, suggesting that rhFGF21 directly acts on astrocytes and may cause some functional alterations. This “reactive profile” identified by *Zamanian* et al. [57] describes the transcriptional programs in astrocytes in response to neuroinflammation (LPS) or ischemia, reflecting changes in morphology, gene expression, and most importantly, functions. In contrast to A1 astrocytes, which express high levels of classic complement cascade genes and are destructive to synapses, A2 astrocytes support neuronal and synaptic survival, rebuild lost synapses and participate in the regulation of neurodevelopmental CNS recovery in neurodegenerative disease by producing neurotrophic factors [58]. RNA sequencing and qRT-PCR analysis showed that rhFGF21-treated astrocytes exhibited notable upregulation of the genes *Clcf1*, *Tgm1*, and *Ptgs2* in the “A2-specific” cluster and the neurotrophic cytokines genes *Bdnf*, *Ngf*, *Fgf1*, and *Fgf2*.

Here, we specifically focused on the possible involvement of the neurotrophins BDNF and NGF, which are upregulated by treatment with rhFGF21 in vitro and elevated in the perilesional cortex in rhFGF21-treated mice. NGF, the first discovered and well-characterized neuropeptide, plays a critical role in regulating the growth and differentiation of cholinergic neurons [59, 60]. NGF can promote neurite outgrowth and cell survival after ischemic injury [61], and deprivation of transported NGF results in damage to nervous cells [62]. BDNF plays a pivotal role in supporting neuronal survival, function, and structural plasticity [63]. Increased BDNF levels in the brain following focal ischemia commonly indicate better recovery and blocking BDNF induction completely negates the recovery of motor function, supporting the critical role of endogenous BDNF in promoting recovery [64–66]. Exogenous administration of BDNF has also been shown to promote functional recovery by increasing axonal sprouting and facilitating synaptogenesis and neurogenesis [67–69]. Therefore, we deduce that rhFGF21 may protect neurons against ischemic injury by acting on astrocytes and producing BDNF and NGF, such as potassium 2-(1-hydroxypentyl)-benzoate [70]. Indeed, in cocultures with hypoxic-ischemic neurons, rhFGF21-treated astrocytes promoted neuronal survival and synaptogenesis. However, the signaling mechanisms involved in the upregulation of astrocytic BDNF and NGF levels by FGF21 were not identified in the current study, and further research is needed.

**Fig. 9:**
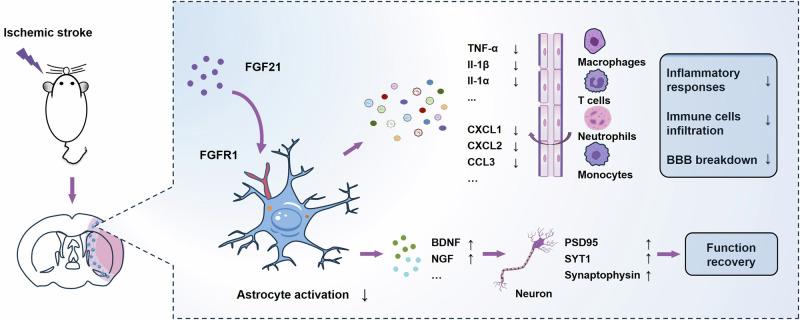
The schematic diagram of the proposed mechanisms regarding the role of FGF21 in ischemic stroke. The neuroprotective effects of FGF21 on ischemic brain injury were achieved through its modulation of astrocyte reactivity, which is mediated mainly by reducing the levels of inflammatory cytokines/chemokines and upregulating the expression of BDNF/NGF.

In summary, FGF21 was identified as an endogenous inhibitor of astrocyte activation with a pronounced impact on ischemic brain injury in an experimental mouse model. We also demonstrated the anti-inflammatory and neurotrophic effects of FGF21 that achieved through its action on astrocytes, which promote neuronal survival and contribute to functional recovery after ischemic injury (Fig. 9). These data present new possibilities for the experimental regulation of astrocyte reactivity and the development of efficient therapeutic approaches for ischemic stroke and other neurological disorders.

## Supplementary information

Supporting information

Supplementary Data
