# Supplementary material for: FGF21, a modulator of astrocyte reactivity, protects against ischemic brain injury through anti-inflammatory and neurotrophic pathways
Source: Acta Pharmacol Sin. 2025 Feb 28;46(7):1834–51. doi: 10.1038/s41401-024-01462-x (PMC12205086; doi:10.1038/s41401-024-01462-x)

Supporting Information for

Original article

**FGF21, as a modulator of astrocyte reactivity, protects against ischemic brain injury through anti-inflammation and neurotrophy pathway**

Dongxue Wang ^1, †^, Wenting Huang ^1, †^, Junfeng Shi ^1^, Fei Liu ^1^, Wenyi Jiang ^1^, Keyang Chen ^1^, Shuyang Zhang^1^, Xiaokun Li ^1*^, Li Lin ^1, *^

^1^ School of Pharmaceutical Sciences, Wenzhou Medical University, Wenzhou, Zhejiang, 325035, China.

^†^These authors contributed equally to this work.

**^⁎^ Corresponding authors:**

**Li Lin.** School of Pharmaceutical Sciences, Wenzhou Medical University, Wenzhou, Zhejiang, 325035, China. E mail: linli@wmu.edu.cn; Tel.: 86-577-86689743.

**Xiaokun Li.** School of Pharmaceutical Sciences, Wenzhou Medical University, Wenzhou, Zhejiang, 325035, China. E mail: [xiaokunli@wmu.edu.cn](mailto:xiaokunli@wmu.edu.cn);

**Other co-authors E-mail addresses:** Dongxue Wang: [dongxue0326@163.com](mailto:dongxue0326@163.com); Wenting Huang: huangwentingwz@163.com; Junfeng Shi: sjf106800@163.com; Fei Liu: [shimfey@163.com](mailto:shimfey@163.com); Wenyi Jiang: 1137133791@qq.com; Keyang Chen: [chenky128@126.com](mailto:chenky128@126.com); Shuyang Zhang: zsynb6662025@163.com;

**1. Supporting figures**

**Fig. S1–S7**


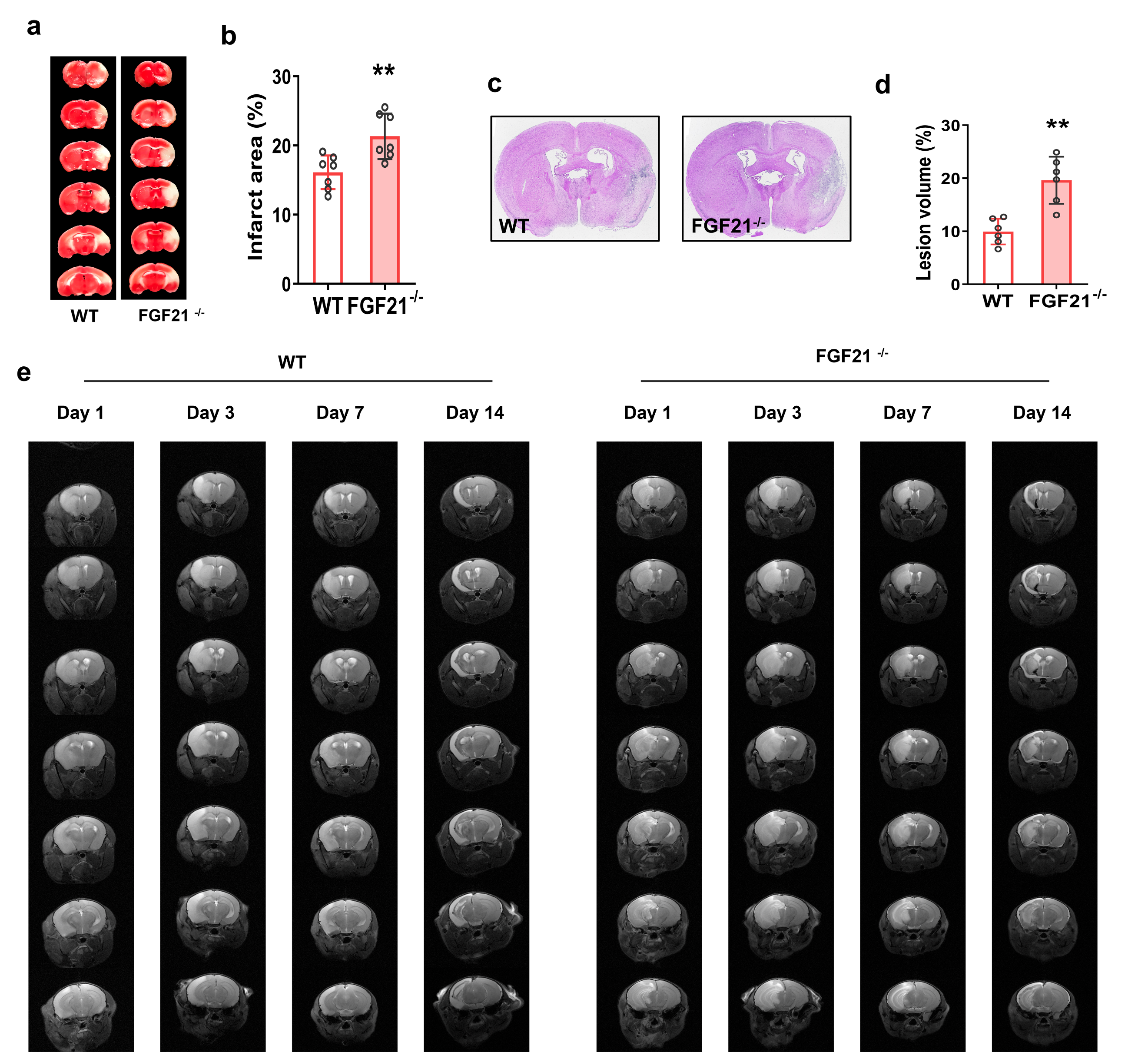


**Fig. S1** Augmented cerebral infarct volume in FGF21^-/-^ mice. **a, b** Representative photographs (**a**) of TTC staining and quantitative data (**b**) of cerebral infarct volume of WT and FGF21^-/-^ mice 3 d after tMCAO. n=8/group. ^**^P<0.001 for FGF21^-/-^ vs. WT group (t-test). **c** The lesion volumes of WT and FGF21^-/-^ mice at 14 d after tMCAO were determined by HE staining. **d** Quantification of brain atrophy by volume. n=6/group. ^**^P<0.001 for FGF21^-/-^ vs. WT group (t-test). **e** Representative sample of 7 consecutive coronal brain sections of WT and FGF21^-/-^ mice at 1, 3, 7, and 14 d post-tMCAO. Scale bar: 0.7 mm.


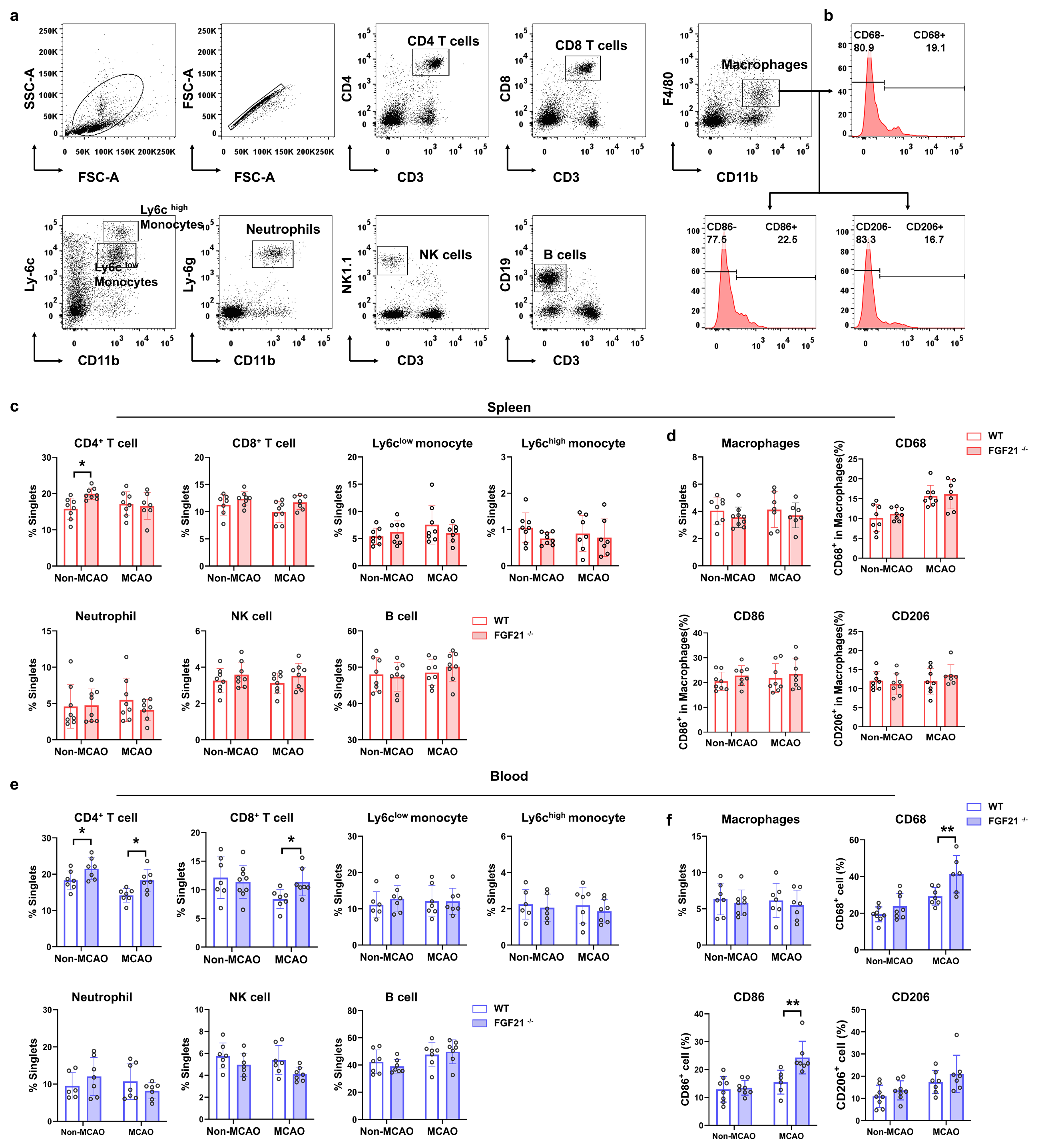


**Fig. S2** Immune cell composition in the spleen and blood of WT and FGF21^-/-^ mice for 3 d post-injury. **a** Representative Dot plots depict the gate strategy of CD4^+^ T cells (CD45^+^CD11b^+^CD3^+^CD4^+^), CD8^+^ T cells (CD45^+^CD11b^+^CD3^+^CD8^+^), macrophages (CD45^+^ CD11b^+^F4/80^+^), neutrophils (CD45^+^CD11b^+^Ly6G^+^), monocytes (CD45^+^CD11b^+^Ly6G^-^Ly6C^low^ and CD45^+^CD11b^+^Ly6G^-^Ly6C^high^), and NK cells (CD45^+^CD3^-^ NK1.1^+^) in spleen and blood. **b** Gate setting for CD86-, CD68-, and CD206-expressing macrophages among the subset of CD45^+^CD11b^+^F4/80^+^. **c, e** Quantification analysis of the indicated immune cells in the spleen (c) and blood (d) tissue from WT and FGF21^-/-^ mice receiving sham- or tMCAO-surgery. **d, f** The composition of macrophages in the spleen (**d**) and blood (**f**) and the percentage of CD86^+^, CD68^+^, and CD206^+^ cells in macrophages were also determined by FACS analysis. n=8-10/group, ^*^P<0.05, ^**^P<0.01, determined by 2way-ANOVA with Sidak’s test.


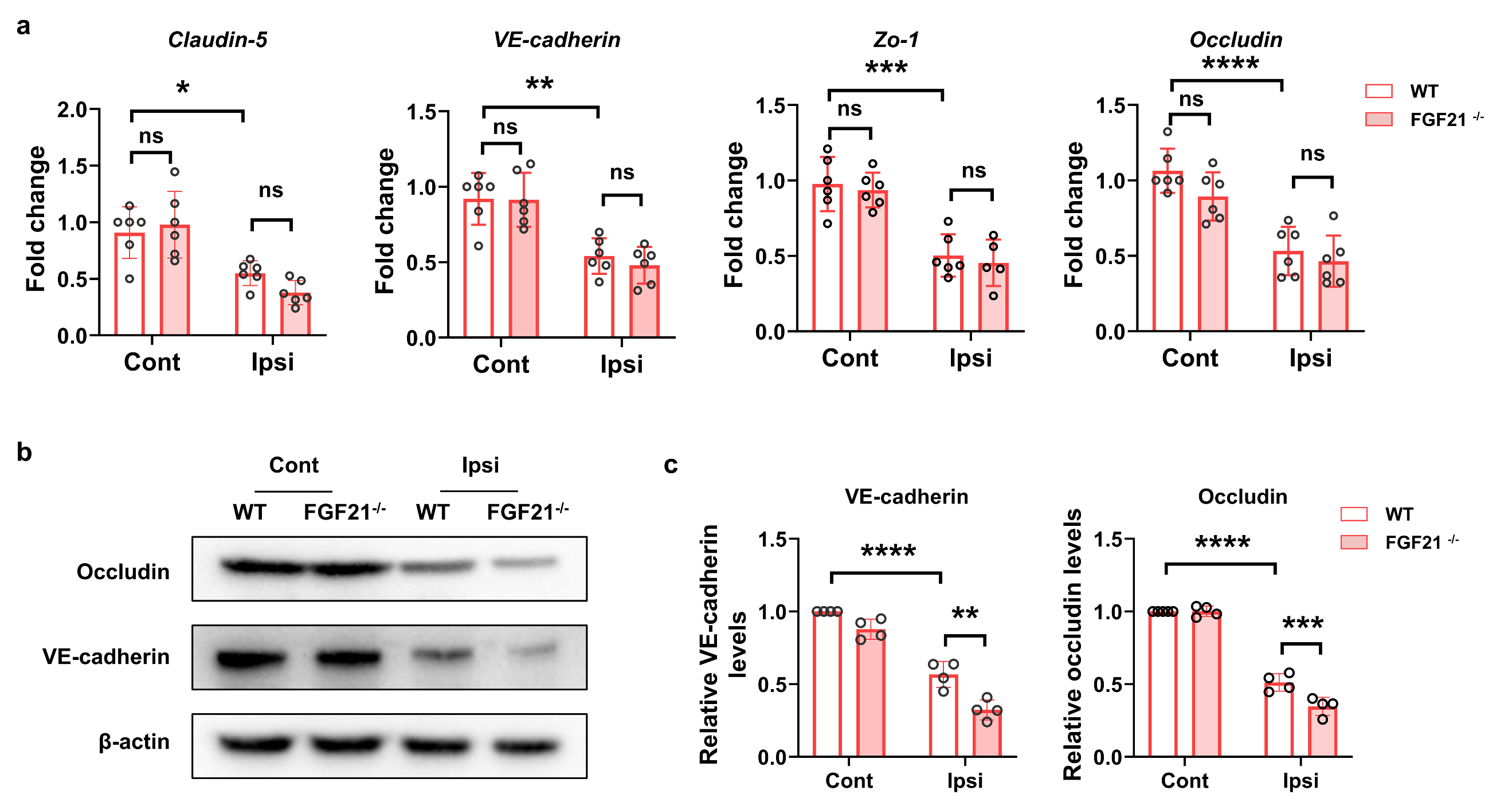


**Fig. S3** Evaluation of BBB breakdown. **a** FGF21 deficiency does not affect the mRNA levels of tight junction-related proteins (*Claudin-5*, *VE-cadherin*, *ZO-1*, and *Occludin*) in the isolated cerebral microvascular fragments, n=6/group. **b** Representative Western blot images and protein expression of Occludin and VE-cadherin in the isolated cerebral microvascular fragments, using β-actin as an internal control. n=4/group, ^*^P<0.05, ^**^P<0.01, ^***^P<0.001, and ^****^P<0.0001, determined by 2way-ANOVA with Tukey’s test.


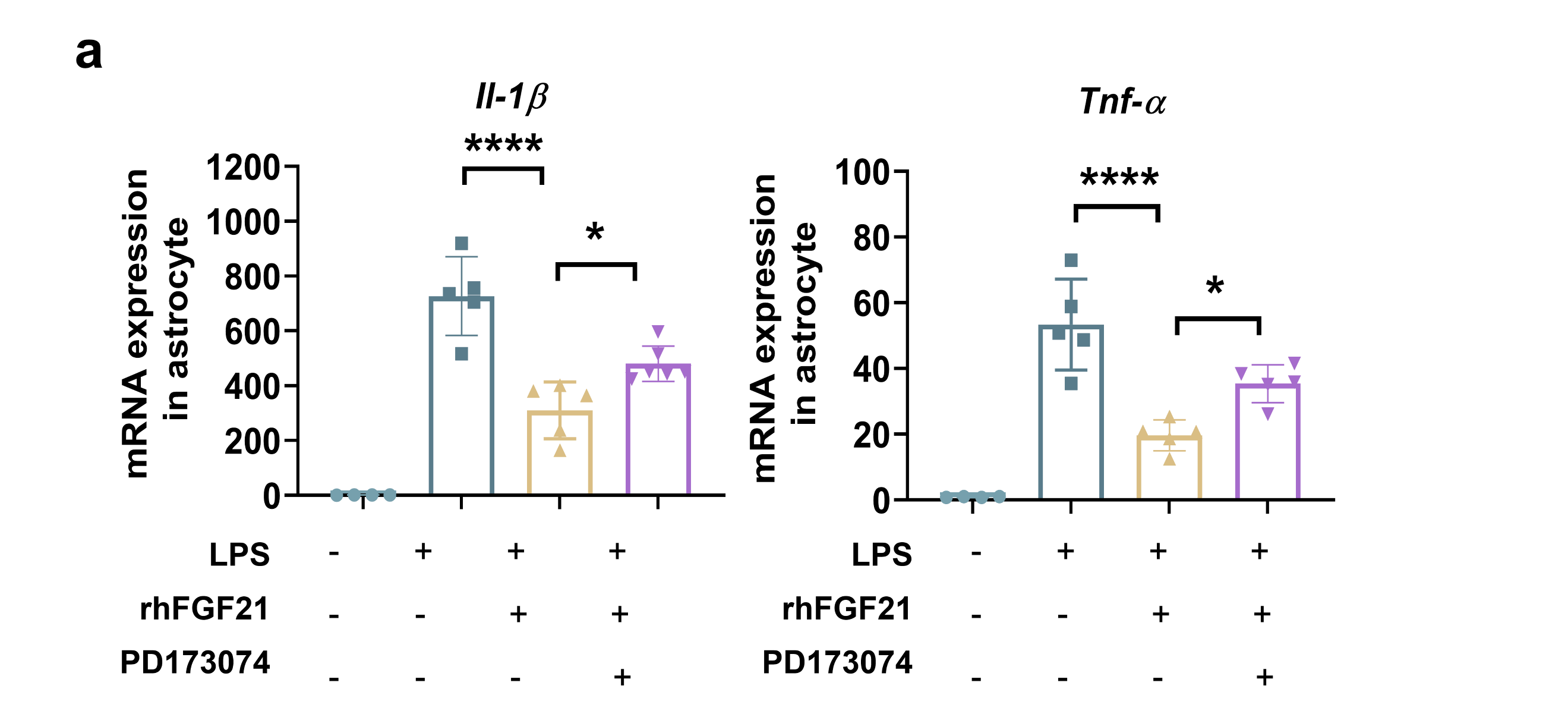


**Fig. S4** Effect of rhFGF21 on LPS-stimulated astrocytes. **a** Primary cultured astrocytes were exposed to LPS stimulates (200 ng/mL) plus vehicle, rhFGF21 (100 nM), or PD173074 (10 μM), and subsequently subjected to quantitative real-time PCR to detect the mRNA level of proinflammatory cytokines of *Il-1β* and *Tnf-α*. n=6. ^*^P<0.05, and ^****^P<0.0001, determined by One-way ANOVA with Tukey’s test.


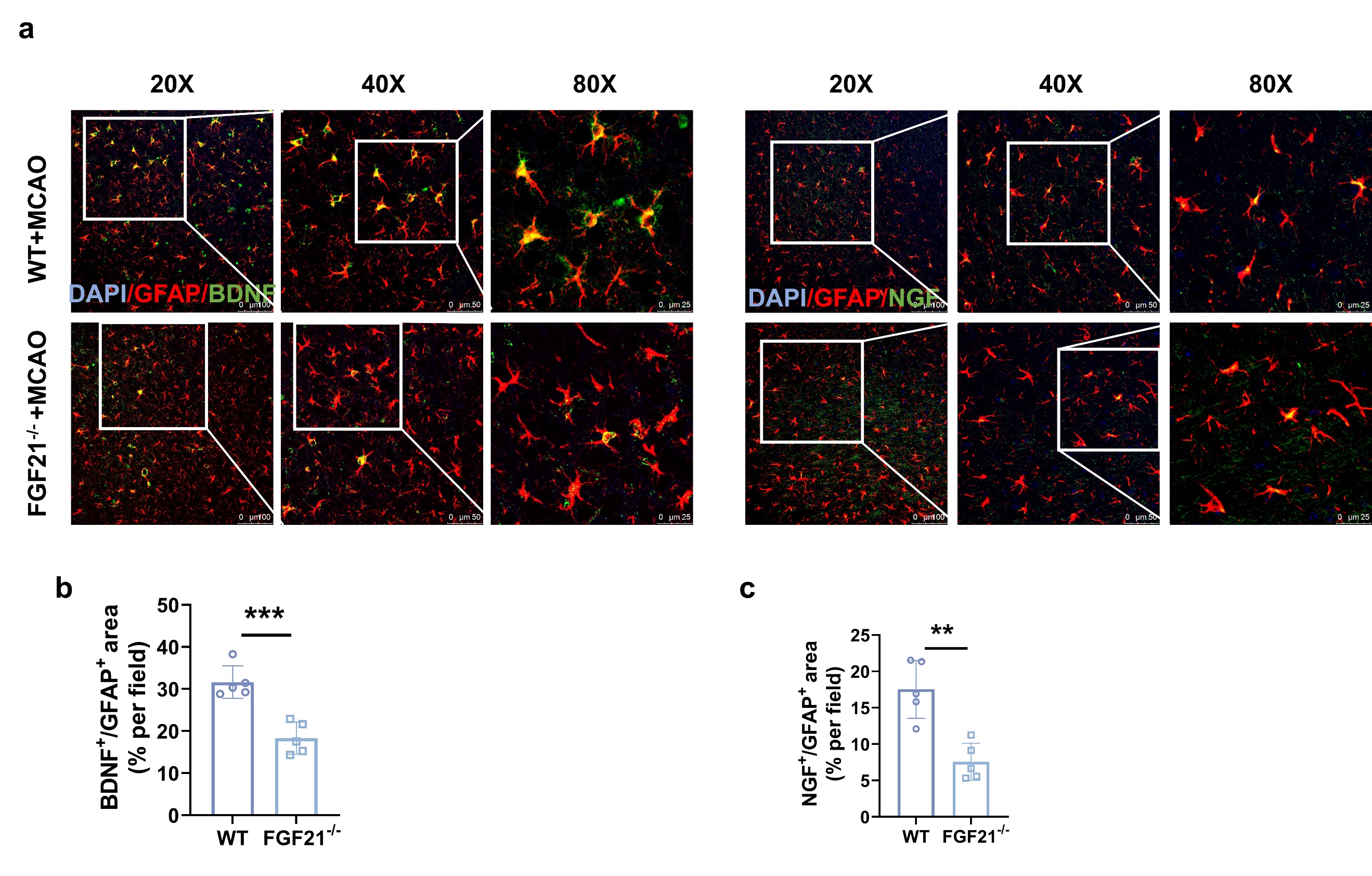


**Fig. S5** Decreased expression of astrocytic BDNF and NGF in FGF21^-/-^ mice. **a** Confocal microscopy images of GFAP-labelled astrocytes (red) and BDNF (green)/NGF (green), in the cortical region adjacent infarct zone. **b-c** Quantified graph show the percentage of BDNF^+^/GFAP^+^ (**b**) and NGF^+^/GFAP^+^ (**c**) cells in the indicated field in WT and FGF21**^-/-^** mice. n=5. ^**^P<0.01, and ^***^P<0.001, determined by t-test.


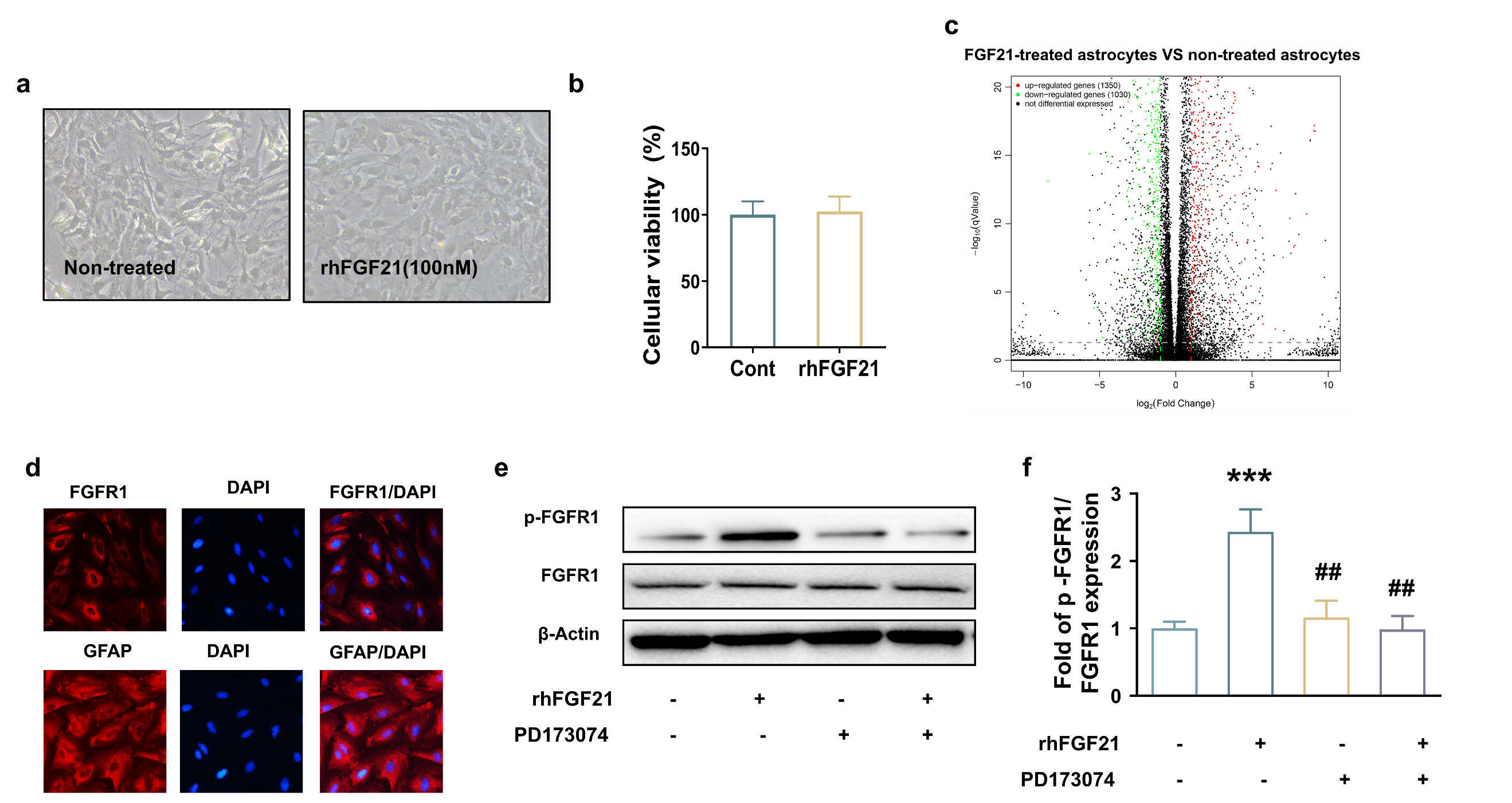


**Fig. S6** Effects of rhFGF21 on resting astrocyte. **a, b** Morphology and astrocytic viability after treatment with 100 nM rhFGF21. **c** Volcano plot of the differentially expressed genes (q Value < 0.05, and Fold change > 2) between FGF21- and non-treated astrocytes. The red dots represent significantly upregulated genes, and the green dots represent downregulated genes. **d** Immunostaining of GFAP and FGFR1 shows the expression of FGFR1 in primary astrocytes. **e, f** The expression of FGFR1 and p-FGFR1was determined by Western blotting after treatment with rhFGF21 and PD173074 - a selective inhibiter of FGFR1. ^***^P<0.001 vs. non-treated astrocytes; ^##^P<0.01 vs. rhFGF21-treated astrocytes (One-way ANOVA with Tukey’s test).


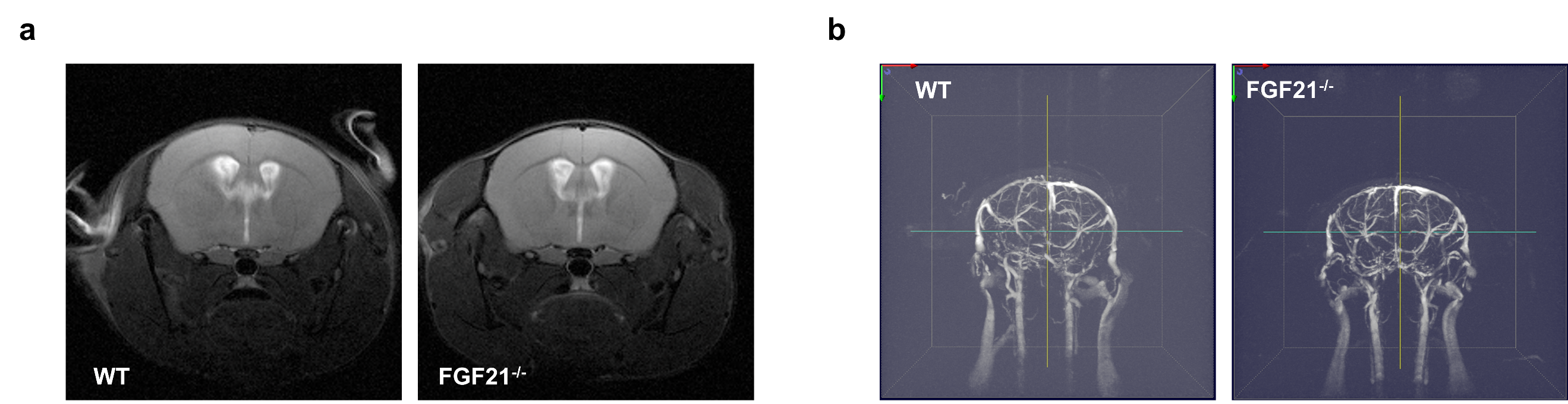


**Fig. S7** Characterization of FGF21**^-/-^** mice. **a** Representative T2-weighted images showing no obviously pathological alterations in brain tissue. **b** MRI of brain vasculature shows no significant alterations of cerebral vasculatures in FGF21**^-/-^** mice.

**2. Supporting table**

**Table S1 Clinical characteristics of human subjects**

|  | Age (yr) | | Gender | Weight | Clinical Diagnosis | NIHSS |
| --- | --- | --- | --- | --- | --- | --- |
| Healthy Subjects | |  |  |  |  |  |
| 1 | 41 | | Male | 72 | NO | / |
| 2 | 47 | | Female | 48 | NO | / |
| 3 | 53 | | Male | 69 | NO | / |
| 4 | 43 | | Female | 54 | NO | / |
| 5 | 45 | | Male | 43 | NO | / |
| 6 | 48 | | Male | 83 | NO | / |
| 7 | 32 | | Male | 82 | NO | / |
| 8 | 37 | | Male | 76 | NO | / |
| 9 | 41 | | Female | 59 | NO | / |
| 10 | 40 | | Female | 45 | NO | / |
| 11 | 67 | | Female | 58 | NO | / |
| 12 | 46 | | Male | 72 | NO | / |
| 13 | 46 | | Male | 72 | NO | / |
| Stroke |  | |  |  |  |  |
| 1 | 66 | | Male | 74 | YES | 9 |
| 2 | 47 | | Male | 73 | YES | 3 |
| 3 | 68 | | Female | 53 | YES | 3 |
| 4 | 71 | | Male | 56 | YES | 15 |
| 5 | 46 | | Male | 66 | YES | 3 |
| 6 | 55 | | Male | 68 | YES | 2 |
| 7 | 53 | | Male | 70 | YES | 2 |
| 8 | 79 | | Male | 59 | YES | 18 |
| 9 | 66 | | Female | 68 | YES | 3 |
| 10 | 79 | | Male | 52 | YES | 18 |
| 11 | 49 | | Male | 78 | YES | 5 |
| 12 | 73 | | Male | 70 | YES | 2 |
| 13 | 69 | | Female | 63 | YES | 1 |
| 14 | 58 | | Male | 65 | YES | 5 |
| 15 | 58 | | Male | 51 | YES | 3 |
| 16 | 80 | | Male | 65 | YES | 2 |
| 17 | 81 | | Female | 55 | YES | 7 |
| 18 | 73 | | Female | 55 | YES | 15 |
| 19 | 67 | | Male | 79 | YES | 1 |
| 20 | 66 | | Male | 75 | YES | 3 |
| 21 | 50 | | Female | 54 | YES | 3 |
| 22 | 79 | | Male | 79 | YES | 1 |
| 23 | 68 | | Male | 55 | YES | 1 |
| 24 | 47 | | Male | 65 | YES | 12 |
| 25 | 59 | | Male | 55 | YES | 2 |
| 26 | 73 | | Female | 61.5 | YES | 2 |
| 27 | 59 | | Female | 75 | YES | 4 |
| 28 | 78 | | Male | 65 | YES | 17 |
| 29 | 80 | | Female | 61.5 | YES | 27 |
| 30 | 83 | | Male | 60 | YES | 2 |
| 31 | 72 | | Male | 68 | YES | 2 |

**3. Uncropped blots of representative bands:**


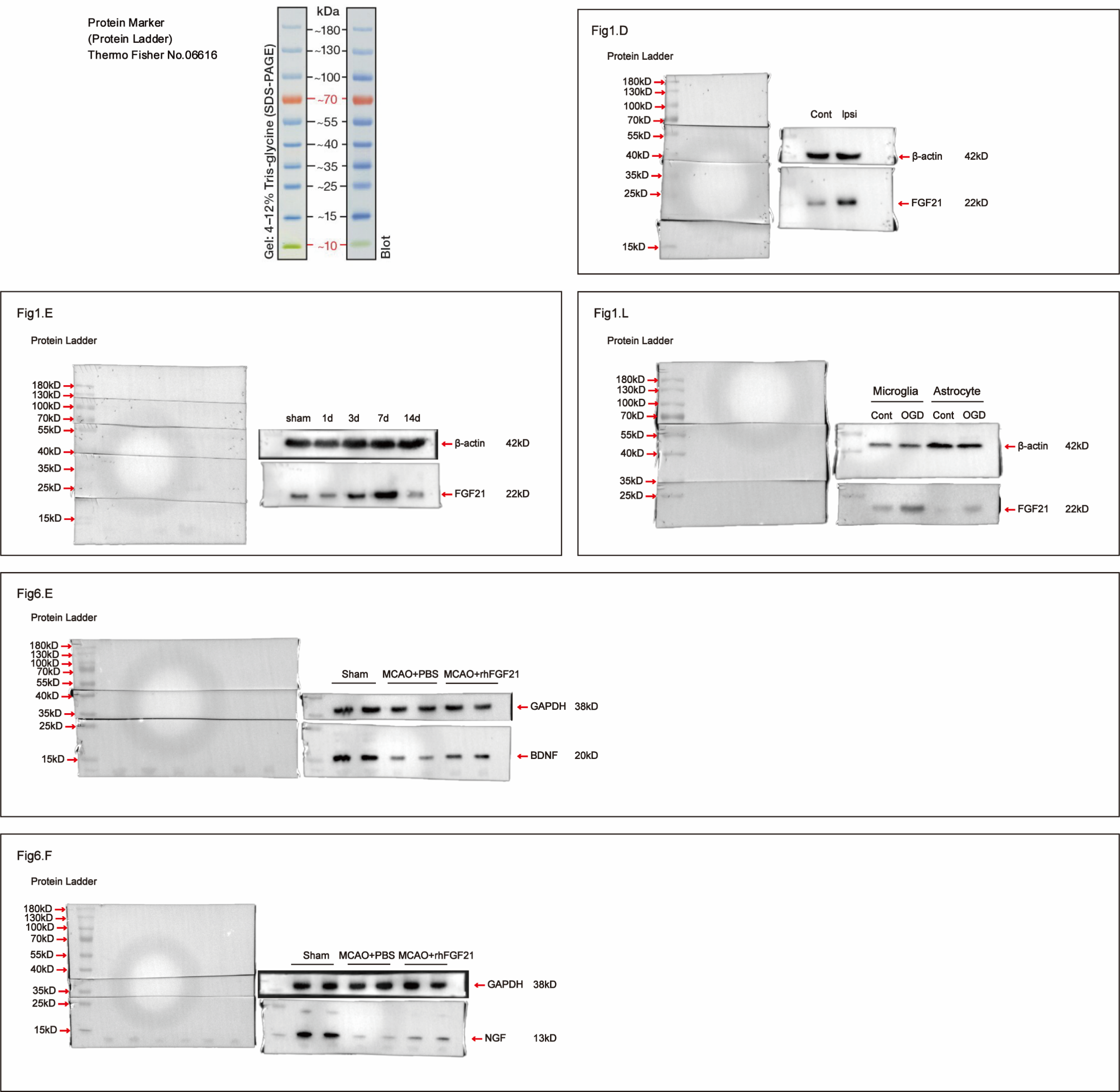


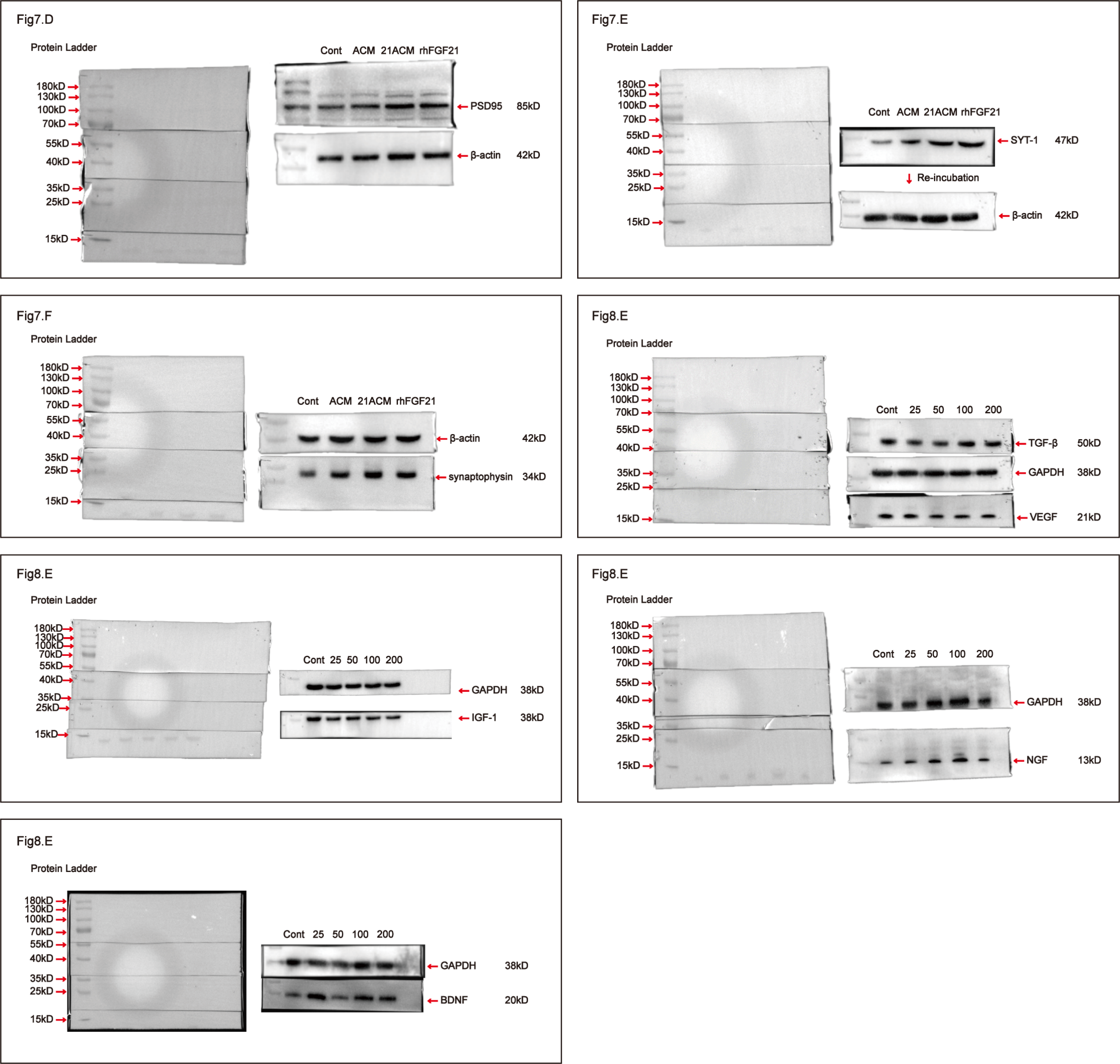

Supplement: Supplementary file 1 — Supporting information [file 41401_2024_1462_MOESM1_ESM.docx]
